# Hydrodynamical Fingerprint of a Neighbour in a Fish Lateral Line

**DOI:** 10.3389/frobt.2022.825889

**Published:** 2022-02-11

**Authors:** Gen Li, Dmitry Kolomenskiy, Hao Liu, Benjamin Thiria, Ramiro Godoy-Diana

**Affiliations:** ^1^ Center for Mathematical Science and Advanced Technology, Japan Agency for Marine-Earth Science and Technology (JAMSTEC), Yokohama, Japan; ^2^ Center for Design, Manufacturing and Materials (CDMM), Skolkovo Institute of Science and Technology, Moscow, Russia; ^3^ Graduated School of Engineering, Chiba University, Chiba, Japan; ^4^ Laboratoire de Physique et Mécanique des Milieux Hétérogènes (PMMH), CNRS UMR 7636, ESPCI Paris—PSL University, Sorbonne Université, Université de Paris, Paris, France

**Keywords:** sensing, surface stress, swimmer interactions, fish school, computational fluid dynamics (CFD), fast fourier transform (FFT), lateral line

## Abstract

For fish, swimming in group may be favorable to individuals. Several works reported that in a fish school, individuals sense and adjust their relative position to prevent collisions and maintain the group formation. Also, from a hydrodynamic perspective, relative-position and kinematic synchronisation between adjacent fish may considerably influence their swimming performance. Fish may sense the relative-position and tail-beat phase difference with their neighbors using both vision and the lateral-line system, however, when swimming in dark or turbid environments, visual information may become unavailable. To understand how lateral-line sensing can enable fish to judge the relative-position and phase-difference with their neighbors, in this study, based on a verified three-dimensional computational fluid dynamics approach, we simulated two fish swimming adjacently with various configurations. The lateral-line signal was obtained by sampling the surface hydrodynamic stress. The sensed signal was processed by Fast Fourier Transform (FFT), which is robust to turbulence and environmental flow. By examining the lateral-line pressure and shear-stress signals in the frequency domain, various states of the neighboring fish were parametrically identified. Our results reveal that the FFT-processed lateral-line signals in one fish may potentially reflect the relative-position, phase-differences, and the tail-beat frequency of its neighbor. Our results shed light on the fluid dynamical aspects of the lateral-line sensing mechanism used by fish. Furthermore, the presented approach based on FFT is especially suitable for applications in bioinspired swimming robotics. We provide suggestions for the design of artificial systems consisting of multiple stress sensors for robotic fish to improve their performance in collective operation.

## Introduction

Collective behavior has been proposed to benefit animals in many aspects, such as predator avoidance, feeding, reproduction, migration and social learning (Partridge, 1982; Pavlov and Kasumyan, 2000; Vicsek and Zafeiris, 2012). Particularly in fish, collective behavior has been recognized as a means to improve energetic performance in swimming (Weihs, 1973; Abrahams and Colgan, 1985; Chen et al., 2016; Ashraf et al., 2017; Filella et al., 2018).

In a fish school, relative position and kinematic synchronisation between neighboring fish are major factors that influence the energetic expenditure of individual fish. Weihs proposed that a diamond-shaped formation would enable one fish to take advantage of the low relative speed passage between the vortex streets formed by two preceding fish (Weihs, 1973). Observations on sea bass by Herskin and Steffensen suggest that swimming at the front of a school was significantly costlier than swimming at the rear (Herskin and Steffensen, 1998). Marras et al. reported that in a fish school, individuals in any position had reduced energetic expenditure: fish swimming behind their neighbors save the most energy, and those fish swimming ahead of their nearest neighbor gain a minor net energetic benefit over swimming solely (Marras et al., 2015). Ashraf et al., by contrast, suggested that a simple “phalanx” formation (swimming in a line) may provide a significant energetical benefit (Ashraf et al., 2017). The choice of relative phase is associated with the relative position between fish: preference of synchronization between nearby fish (in-phase or anti-phase) is observed in fish swimming experiments (Ashraf et al., 2016, 2017), while the beneficial outcome of synchronization are confirmed numerically (Li et al., 2019a) and experimentally (Godoy-Diana et al., 2019). Besides synchronization, recent studies also suggest that matching the vortex phase of a neighboring fish is an effective means to improve the energetic efficiency (Daghooghi and Borazjani, 2015; Khalid et al., 2016; Li et al., 2020). In that case, the optimal phase between neighbors is dynamic and should be adjusted according to their relative position and wake morphology.

Effective sensing is important to maintain the group configuration (Puckett et al., 2018). The sensory basis of fish schooling includes multiple systems such as the lateral line and vision (Partridge and Pitcher, 1980; Partridge, 1981). Attenuated vision may alter schooling behavior: fish are observed to swim slower in low light environment (Berdahl et al., 2013), and keep larger relative distance (Hunter, 1968). Nevertheless, fish can school even under a blindfolding condition, while blinding had little effect on the position that experimental fish took up with respect to their neighbors within the school (Pitcher et al., 1976; Partridge and Pitcher, 1980). This indicates the significant role of non-visual sensing in fish schooling behavior. On the other hand, ablating the lateral-line also alters schooling behavior (Partridge and Pitcher, 1980; Faucher et al., 2010; Mekdara et al., 2018). Fish may still school when the posterior lateral-line system is disabled, but they reduce the distance to neighboring fish and may occasionally collide (Partridge and Pitcher, 1980). A more recent study reports that, without functioning superficial neuromasts, schooling behavior was disrupted under both photopic and scotopic conditions and the ability to detect stationary objects decreased (Middlemiss et al., 2017). Experiment on rummy-nose tetra fish shows that, when the whole lateral-line system is inactivated, fish cannot maintain the schooling behavior and swim with greater distances between neighbors (Faucher et al., 2010). Mekdara et al. found that tail beat synchronization during schooling requires a functional posterior lateral-line system in Giant Danios, and they hypothesize that the anterior branch may be more important for regulating position within the school, while the posterior branch may be more important for synchronizing tail movements (Mekdara et al., 2021).

Lateral-line sensing is realized by a mechanosensory system, comprised of arrays of sensors called neuromasts, which respond to the motion of the surrounding water relative to the skin of the fish, and to pressure and shear stress changes (Coombs et al., 1988; Montgomery et al., 1995; Coombs, 2001; Ren and Mohseni, 2012). There are two main types of lateral-line organs in lower vertebrates: superficial neuromasts (SN), with a cupula that protrudes in the surrounding water, and canal neuromasts (CN), located in the lateral-line canal. The scales of the trunk lateral-line canal of fish contain SNs as well as CNs (Kroese and Schellart, 1992; Coombs, 2001). While the neural response of the hair cell is proportional to the displacement of the cupula and the underlying ciliary bundles (van Netten and Kroese, 1987), cupula displacement is largely proportional to the velocity of water flowing past it, coupling the motion of the surrounding water to the underlying cilia through viscous forces. On the other hand, inertial forces are required for the fluid to break through the boundary layer and move into the small-diameter canal. Hence, flow velocity inside the canal is more or less proportional to the net acceleration between the fish and the surrounding water, CNs respond to changes in external flow acceleration and to net pressure differences between the two surrounding canal pores (Denton and Gray, 1983; Kroese and Schellart, 1992)**.** CNs and SNs may have different response characteristics: SNs best respond to the direct current and low-frequency components of the incoming flow, whereas CNs respond best to high-frequency components of the flow (Coombs et al., 2001).

Artificial lateral lines with biomimetic neuromasts have been developed and applied to bioinspired swimming robots (Fan et al., 2002; Yang et al., 2010; Xu and Mohseni, 2017; Liu et al., 2020). Optimal sensor locations for artificial swimmers have been also investigated to improve the efficiency (Verma et al., 2019; Weber et al., 2020), especially when the number of biomimetic neuromasts is limited. The sensing of external hydrodynamic stress may improve the control robustness in single-fish self-organized undulatory swimming (Thandiackal et al., 2021), as well as the propulsive efficiency (Tytell et al., 2010). In collective swimming, the interaction of the flow field and the surface stress between neighboring fish have been established by several recent studies (Daghooghi and Borazjani, 2015; Chen et al., 2016; Peng et al., 2018, Li et al., 2019a; 2019b; Verma et al., 2019). This interaction is the basis for perceiving the collective swimming configuration by stress-sensing. It is reasonable to expect that fish tune the collective swimming spatio-temporal patterns and improve the collective swimming efficiency based on stress information (Novati et al., 2017; Verma et al., 2018).

The aim of this study is to investigate, using a numerical model, the stress signal features induced by a nearby swimming fish, and examine how to identify the state of a neighbor fish based on lateral-line sensing. The general objective is to assess to what extent the sensing of flow fluctuations through lateral line sensors can be used to determine decision making in schooling fish. Since fish deformation and deformation-induced stress are periodic, we apply a Fourier Transform to identify the signal changes with respect to different frequency components. The zero-frequency component (constant in Fourier Transform), the tail-beat frequency component, and the higher-frequency ones correspond, respectively, to large-scale environmental flow, to the fish own tail-beat behavior, and to turbulence. Previous biological studies provide basis for such decomposition: in the fish peripheral nervous system, superficial neuromasts respond best to slow unidirectional flows, whereas canal neuromasts respond best to more rapidly fluctuating flows (e.g., 30-100 Hz); in the fish central nervous system, there is also a filter mechanism to increase signal-to-noise ratios by filtering-out the fish’s own movements (see Coombs, 2001, for a review). This analysis based on Fourier Transform provides a new angle to quantitatively understand the hydrodynamic interaction in collective swimming, and is especially suitable to for implementation in swimming robotic control.

## Methods

### Fish Morphology and Kinematics

We simulated a virtual swimmer with body shape based on the profile of an adult trout (Supplemenatary Figure S12). The body length is set as 4 cm to limit the simulation within laminar flow, and the body wave was prescribed by sinusoidal functions:

$$H\left(l,t\right)=\alpha \cdot l^{2}\cdot \sin\left(\frac{2\text{π}l}{\lambda }-2\text{π}ft\right)$$ (1)

where H(l,t)  is the dimensionless lateral excursion at time t ; α is a factor that used to control amplitude, set as 0.1 in the standard case; l∈[0,1] is the dimensionless distance from the snout along the longitudinal axis; λ is the dimensionless length of the body wave based on body length, set as λ=1.1 to match typical kinematics of carangiform swimmer (Wardle et al., 1995). Eq. 1 causes the total body length along the midline to vary during the tail beat, which is corrected by a procedure that preserves the lateral excursion while ensuring that the body length remains constant (see Supplemenatary Figure S13 in Supplemenatary Materials).

### Three Dimensional Navier-Stokes Solver

The hydrodynamic solution is based on a validated three-dimensional Navier-Stokes (NS) solver (Liu, 2009; Li et al., 2012; 2014; Bomphrey et al., 2017). The governing equations for fluid solution are three-dimensional, incompressible and unsteady NS equations written in strong conservation form for mass and momentum (Liu, 2009). To accelerate the computation and improve the robustness during iteration, the artificial compressibility method is adopted by adding a pseudo time derivative of pressure to the continuity equation. The solving process is implemented in multiple grids using the finite volume method (FVM). Navier-Stokes equations were solved in each grid and results were interpolated at the grid interfaces.

The approach comprises surface models of the changing fish shape (dimension: 133 × 97), and local fine-scale body-fitted grids (dimension: 133 × 97 × 20) plus a large stationary global grid (dimension: various) to calculate the flow patterns around the fish with sufficient resolution. The boundary conditions are set as follows: 1) in the fish-body-fitted grid, the non-slip condition is applied to the cells on the surface of the fish body; 2) in the global grid, an incoming flow U is set for the frontal surface, while a zero-gradient condition is used for other surfaces; 3) at the interfaces between fish-body-fitted and background blocks, the two blocks provide boundary conditions to each other through interpolations.

We implemented pre-simulations on a single fish to determine an equilibrium speed *U* of 2.375*L* s^−1^ (9.5 cm s^−1^), at which the drag balances the propulsion by the fish. In all the simulations in this study, the centers of mass of fish were fixed, while the incoming flow was set as *U*, so that the hydrodynamics of the tethered fish were similar to those in realistic steady swimming. The Reynolds number of the simulations is defined as Re=ρUL/μ , where ρ is the water density, *U* is the swimming speed, *L* is the body length (= 4 cm), and μ is the dynamic viscosity of water. Corresponding to the incoming flow speed, Re was set as approximately 4,300, and no turbulence model was applied in the simulation. Information on the validation of grid resolution, including the radial-direction grid resolution test, is provided in§C of Electronical Supplemenatary Materials.

The computational accuracy in terms of stress and flow around a fluctuating rigid cylinder is validated in §B-8 in the Supplemenatary Materials of (Li et al., 2021)). The validation of flow simulation accuracy around a deforming fish body is provided in Figure 9 and Fig. 18 in (Li et al., 2012), which can also be an indirect validation of stress accuracy on a deforming fish body, since surface stress is coupled with near-surface flow motion. Further details of the NS solver are provided in Part C of Supplemenatary Materials.

### Virtual Lateral Line

We assume that the model fish possesses a continuous virtual lateral line at mid-body height, from snout to tail tip, on both sides of the body. The virtual lateral-line may accurately sense both local pressure- and shear-stresses. The lateral-line stress signal **X** (*s, b, l*) during an arbitrary tail-beat cycle is sampled 40 times (sampling rate = 320 Hz), where *s* represents the stress type (*s* = pressure or shear stress), *b* indicates the body side (*b* = left or right); and *l* represents the sensor position along the body (0 < *l* < *L*).

### Stress Signal Processing by Fast Fourier Transform (FFT)

Due the specific nature of the fish-like swimming kinematics, the pressure- and shear stress-over the fish surface shows strong periodicity: a frequency-domain analysis is thus ideal to examine the lateral-line stress signal. The lateral-line stress signal **X** during an arbitrary tail-beat cycle is sampled and processed by a fast Fourier transform (FFT) algorithm (function: *fft*, MATLAB R2020b, The Mathworks).

Signals are processed and decomposed with respect to the dimensionless frequency *k* used in this study, computed as *k = f*/*f*_ref_, where the *k =* 1 component corresponds to a sinusoidal signal in tail-beat frequency, and *k* = 0 corresponds to the direct current component. We will use in the paper the direct current (DC), alternating current (AC) nomenclature. The single-sided spectrum is computed for all components. Note that for an AC component, the single-sided spectrum only shows its magnitude, while for the DC component, we preserve the sign of signal since the negativity of the DC component represents hydrodynamic information of the stress direction. The phase of each component is computed by the MATLAB function *angle*.

Further details of the Fast Fourier Transform are provided in §C-5 of Electronical Supplemenatary Materials.

## Results

### Basal Signal in a Swimming Fish Alone

**FIGURE 1:**
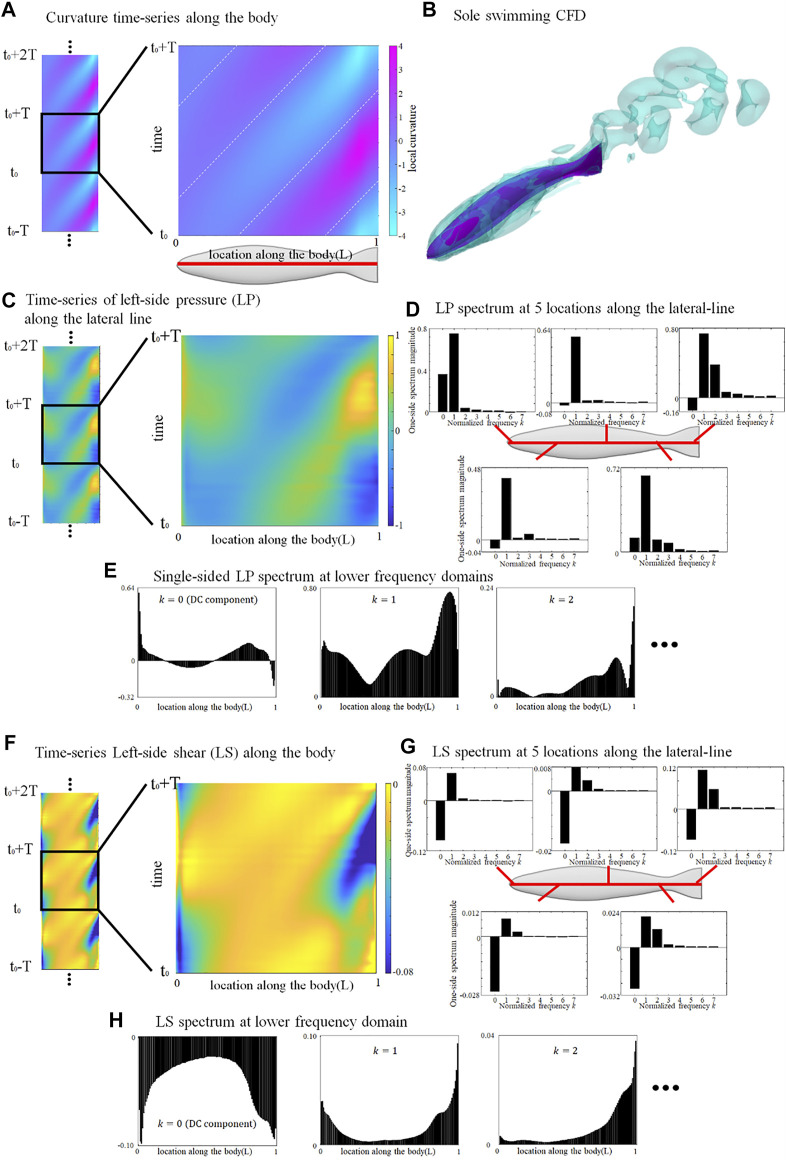
Deformation and lateral-line stress in solely swimming fish. **(A)** curvature map of fish axis in an arbitrary tail-beat cycle (from *t*_0_ to *t*_0_+*T*); **(B)** Flow field surrounding a solely swimming fish; **(C)** pressure-stress map of the left-side lateral-line; **(D)** Frequency domain analysis of pressure stress signal at five locations along the lateral-line; **(E)** Distribution of single-sided spectrum of left-side pressure, at *k* = 0, 1, and 2; **(F)** shear-stress map of the left-side lateral-line; **(G)** Frequency domain analysis of shear-stress signal at five locations along the lateral-line; **(H)** Distribution of single-sided spectrum of left-side shear-stress signal, at *k* = 0, 1, and 2. One stress unit = 10 Pa.

The lateral-line signal of a swimming fish alone provides a basal value to estimate the relative stress change associated to collective swimming. Since the deformation is governed by a sinusoidal function, the curvature time-series periodically repeats a same pattern. We choose an arbitrary tail-beat cycle (from *t*_0_ to *t*_0_+*T*) and the curvature map is demonstrated in Figure 1A.

The left-side lateral-line pressure signal map during one tail-beat cycle is displayed in Figure 1C. The pressure-signal map sensed by the right-side lateral-line is omitted since it is of an identical pattern to the left-side but with a half-tailbeat-cycle phase difference ( Δφ=π ). Based on FFT, in Figure 1D, the period diagram at five positions along the lateral-line is sampled and the magnitude of the single-sided spectrum is expressed by histogram. Figure 1E shows the distribution along the lateral-line of the single-sided spectrum for the three lowest modes ( k = 0, 1, and 2), which are the more energetic ones. The DC signal ( k = 0) is positive and strong in the frontal area of the fish, representing a high frontal pressure due to form drag. The magnitude of the AC signal majorly concentrates in the k  = 1 term (i.e., the frequency equivalent to the tail-beat frequency) and the residual strength can be basically expressed by second and third order terms ( k  = 2 and 3). The k  = 1 AC signal is dominant, and usually higher than the DC signal, suggesting that the instantaneous pressure-stress on the entire body undergoes strong fluctuations associated to the body undulation, and may switch between positive and negative values. We also observe that the signal has a low amplitude location at l≈0.4L .

Figure 1F shows the shear-stress signal map sensed by the left lateral-line from *t*_0_ to *t*_0_+*T*. Unlike the pressure stress that shifts between positive and negative values, the shear-stress signal map is overall negative, indicating that shear stress consistently hinders the forward motion of the fish. The magnitude of the DC signal is usually stronger than the k  = 1 AC signal along the body, except near the tail tip (Figures 1G,H). The strength of the shear-stress signal majorly concentrates in the DC, k  = 1 and k  = 2 AC terms.

**FIGURE 2:**
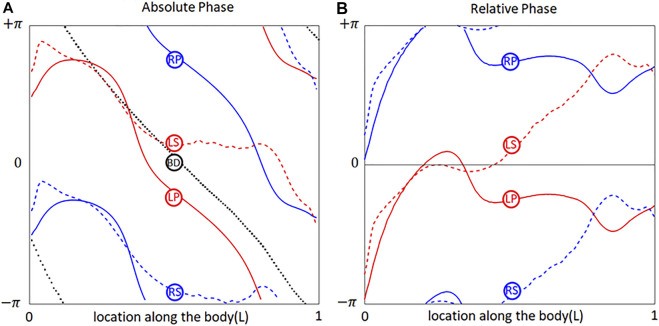
Phases of k = 1 AC component of respectively body deformation (BD), left lateral-line pressure (LP), right lateral-line pressure (RP), left lateral-line shear-stress (LS), right lateral-line shear-stress (RS). **(A)** Absolute values; **(B)** Relative values about the body deformation phase.

Since the k  = 1 component is dominant in the AC signal magnitude, when analyzing the phase of AC signals we only focus on k = 1 component. Its phases of body deformation (BD), left lateral-line pressure (LP), right lateral-line pressure (RP), left lateral-line shear-stress (LS), right lateral-line shear-stress (RS) are shown in Figure 2A. Here, the body deformation (BD) line is straight at first order (although a slight deviation from the straight pattern results from the length correction algorithm that prevents the body elongation caused by Eq. 1). The phase differences between LP and RP, as well as between LS and RS, is a half tailbeat-cycle ( Δφ=π ). The initial values of the absolute phases depend on the choice of *t*_0_. To eliminate this influence of the choice of *t*_0_, we calculated the phases of LP, RP, LS and RS relative to the body deformation (Figure 2B).

### Presence of an Alongside, In-phase Neighbor

**FIGURE 3:**
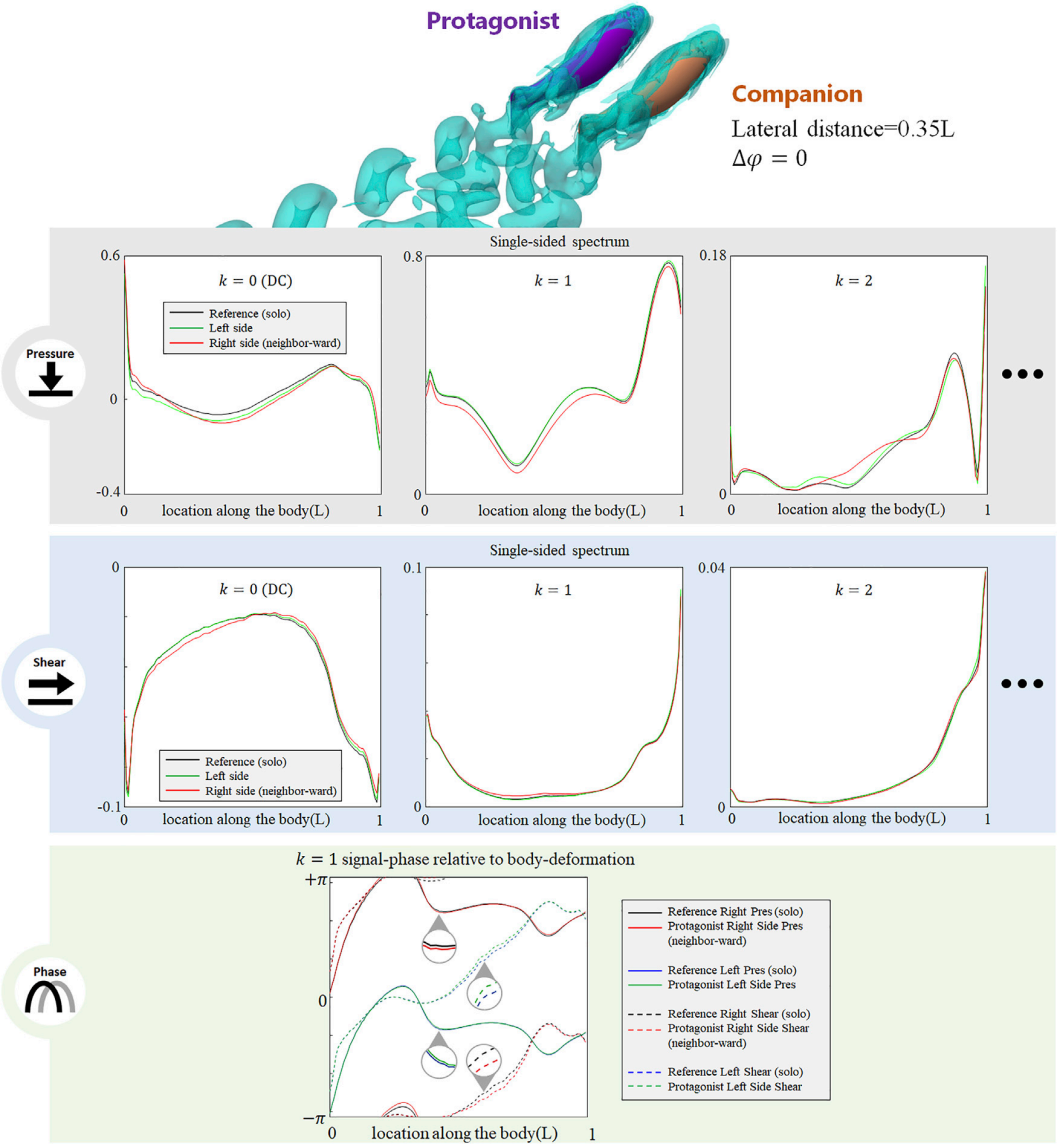
Signal features sensed by the protagonist fish when an alongside, in-phase neighbor is 0.35 L away on its right side. Full results are presented in Supplementary Figure S1, ESM. One stress unit = 10 Pa.

The presence of an alongside neighbor may influence the lateral-line signal in several aspects. Figure 3 shows the signal features sensed by the protagonist fish when an alongside, in-phase neighbor is 0.35 L away on its right side. In terms of pressure stress, the DC term of both left and right sides changed: on the right side (neighbor-ward) the DC pressure signal becomes greater at the head and tail zones, and weakens in the mid-region of the body. On the left side (free side) the DC pressure signal of the anterior body slightly decreases, suggesting that the free side may still be influenced by the presence of the neighbor fish. A similar phenomenon (stress influence across the body) is also reported in Verma et al. (2019). For *k* = 1 AC signal, the pressure on the free side is consistent with the reference signal, while it is overall weak on the neighbor-ward side. The latter is considered as a key feature in this collective swimming configuration. In terms of shear stress, on the neighbor-ward side the shear stress DC signal is reduced in the anterior part and increased in the posterior part. The *k* = 1 AC shear signal shows an increment in the middle of body (0.3–0.5*L*), and since the basal signal is relatively low at this location, there is an approximately 30% increment in proportion.

For conciseness purposes, Figure 3 only includes the most significant features, and full results are presented in Supplemenatary Figure S1, ESM.

### Dependence on the Distance of the Alongside In-phase Neighbor

**FIGURE 4:**
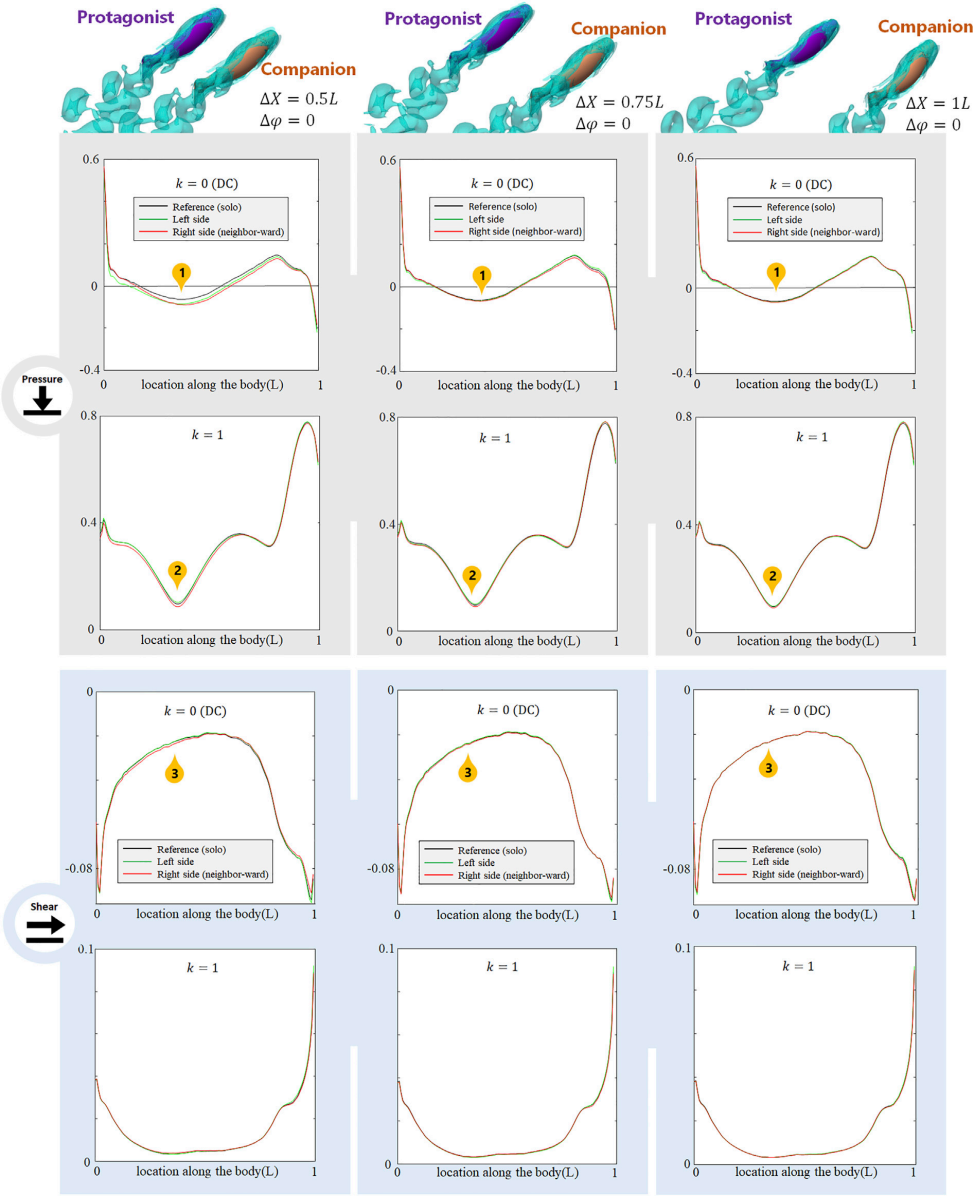
Signal features sensed by the protagonist fish when an alongside, in-phase neighbor is 0.5 L,0.75 and 1 L away on its right side. Full results are presented in Supplementary Figures S2-S4, ESM. One stress unit = 10 Pa.

As the alongside, in-phase ( Δφ=0 ) neighbor moves further from the protagonist fish, the stress influence on the protagonist fish gradually fades out. Figure 4 shows the signal features as the alongside, in-phase neighbor increases its distance ΔX to 0.5, 0.75 and 1 L. The DC pressure difference at Location 1, the *k* = 1 AC pressure difference at Location 2, and the DC shear-stress difference at Location 3 all appear to attenuate. Since the change of stress difference from ΔX=0.35L to 1 L is significant, fish may be able to sense the lateral distance change based on the DC or *k* = 1 AC lateral-line stress. Also, the fish may sense on which side the neighbor approaches, based on the shear stress change at Location 3. However, the neighbor-induced stress influence almost fully fades out as the alongside neighbor fish is more than 1*L* away, and it becomes seemingly difficult to accurately judge the relative location of neighbor fish.

The alongside, in-phase neighbor may also cause changes in higher order (*k* > 2) AC components. These results are shown in Supplemenatary Figure S2-S4, ESM.

### Phase Difference With the Alongside Neighbor

**FIGURE 5:**
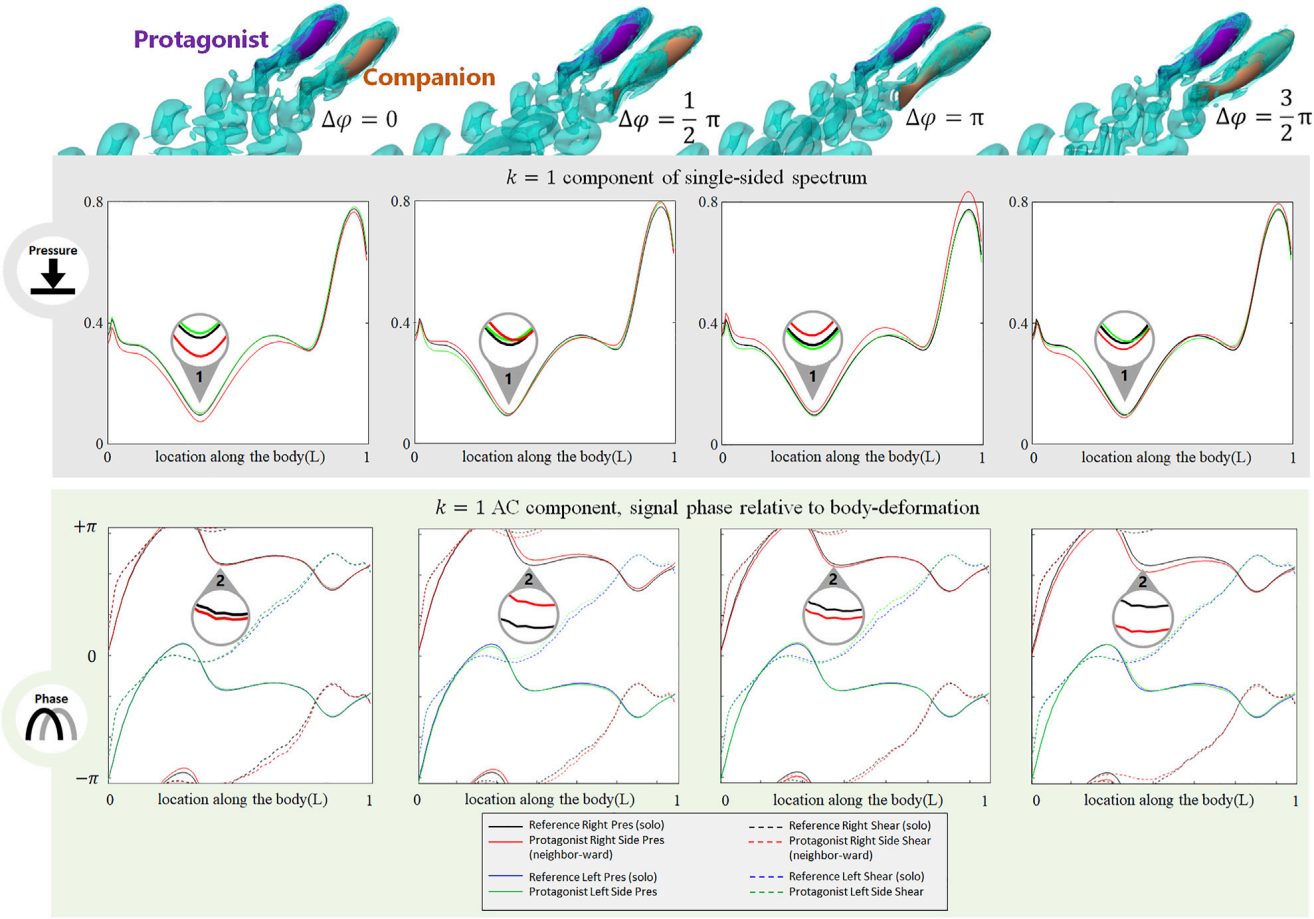
Signal features sensed by the protagonist fish when an alongside neighbor is 0.35 L away on its right side, with $\Delta \varphi =0,\frac{1}{2}\pi$ , $\pi \;\text{and}\frac{3}{2}\pi$ . Full results are presented in Supplementary Figure S1, Supplementary Figures S5,S7, ESM. One stress unit = 10 Pa.

The phase difference between two adjacent fish also induces changes in the stress signal. As shown in Figure 5, when a neighbor fish swims alongside the protagonist with various phase differences $\Delta \varphi =0,\frac{1}{2}\pi$ , $\pi \;\text{and}\frac{3}{2}\pi$ , the *k* = 1 AC pressure signal is most influenced (Location 1): when Δφ=0 , the *k* = 1 AC pressure signal on the neighbor-ward side at all locations along the body is weakened. On the contrary, when Δφ=π , the *k* = 1 AC pressure signal on the neighbor-ward side is strengthened along the body. Under the other two phase-difference conditions, $\Delta \varphi =\frac{1}{2}\pi$ and $\frac{3}{2}\pi$ , the *k* = 1 AC pressure signal on the neighbor-ward side shows a pattern between that of Δφ=0 and π , becoming weakened and strengthened in a staggered pattern.

The phase of the lateral line signal is also sensitive to the phase difference with a neighbor fish. As shown in Figure 5, at Location 2, the *k* = 1 AC pressure signal phase on the neighbor-ward side shifts from the reference (i.e., the gap between the red and black solid lines). The phase-shift is most significant at $\Delta \varphi =\frac{1}{2}\pi$ and $\frac{3}{2}\pi$ . When $\Delta \varphi =\frac{1}{2}\pi$ , the *k* = 1 AC pressure signal on the neighbor-ward side shifts to an advanced phase, while when $\Delta \varphi =\frac{3}{2}\pi$ , it shifts to a delayed phase. When Δφ=0 or π , the signal phase shift is negligible.

In contrast to the neighbor-ward side, the pressure stress signal on the free-side is very similar to the reference value (Figure 5: the difference between the green and blue solid lines is small), suggesting fish may sense a phase shift between its neighbor-ward and free sides, and may further judge the phase of the neighbor fish based on the relative change of magnitude and phase of the *k* = 1 AC pressure signal between its two sides.

In terms of the DC component (for full results, see Supplemenatary Figure S1, Supplemenatary Figure S5-S7, ESM), for both pressure and shear stress at $\Delta \varphi =\frac{1}{2}\pi ,\;\pi$ and $\frac{3}{2}\pi$ , the signal change on the neighbor-ward side is similar to that of the Δφ=0 case, suggesting the DC component change is relatively robust to Δφ .

### Diagonal Configuration

**FIGURE 6:**
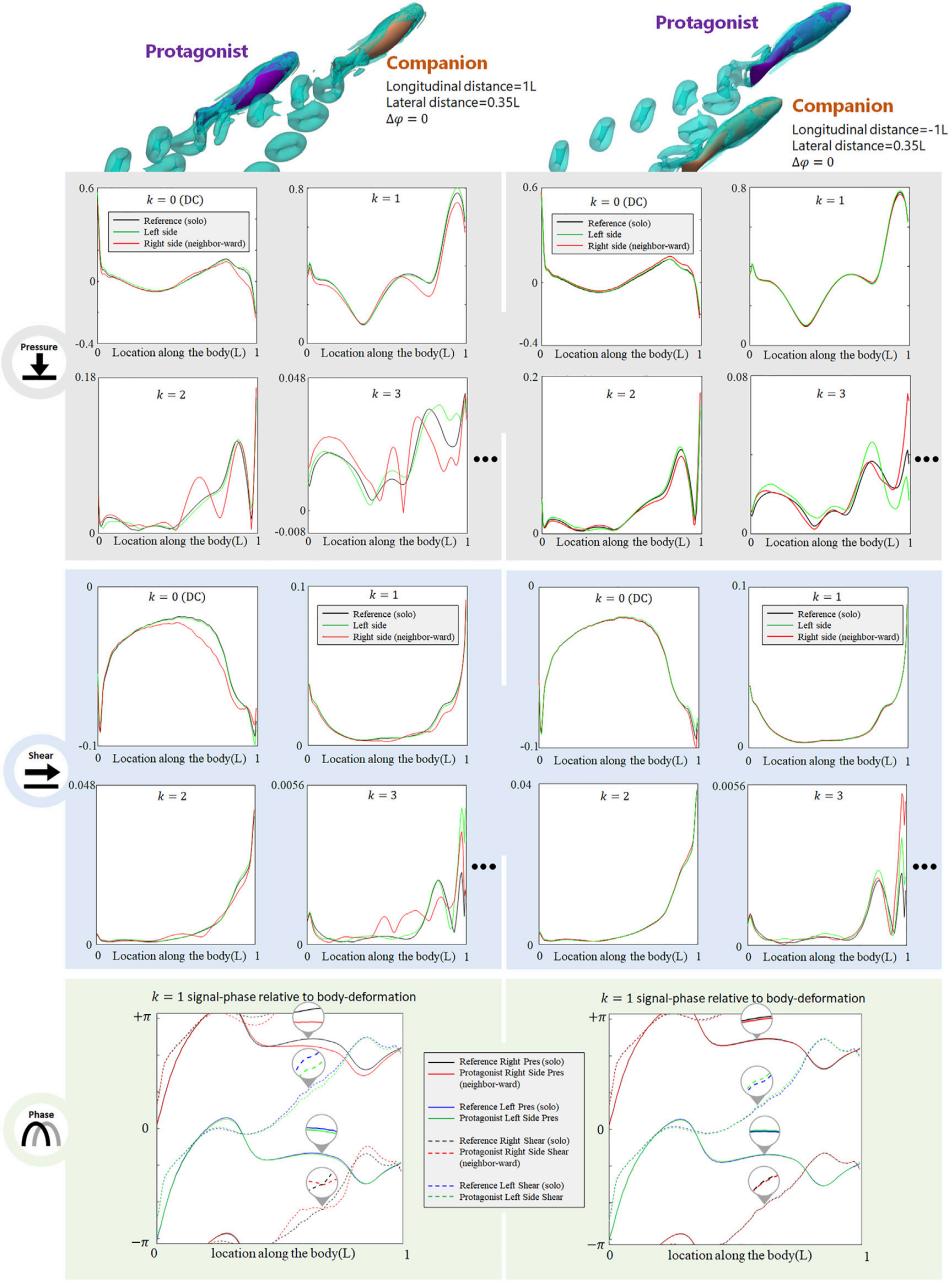
Signal features sensed by the protagonist fish when an in-phase neighbor is in diagonal front position (LHS), and diagonal rear position (RHS). Full results are presented in Supplementary Figures S8,S9, ESM. One stress unit = 10 Pa.

When the neighbor fish is located in the front diagonal of the protagonist fish (see Figure 6, LHS), the posterior body of the protagonist fish is exposed to the wake of the neighbor fish and the protagonist fish may easily sense the associated flow fluctuations. As shown in the LHS of Figure 6, on the neighbor-ward side, both DC and AC components of pressure- and shear-stress signals in the posterior body undergo noticeable changes. For the pressure, the DC and the *k* = 1 and 2 AC components show a magnitude fluctuation mainly in posterior body, while the anterior part signal is less influenced. The *k* = 3 AC components of pressure signal undergo complex magnitude changes along the entire body, with strengthened and weakened zones in a staggered pattern. Concerning the shear-stress signal, the DC and *k* = 1 AC components show a magnitude reduction in the posterior part of the body. The *k* = 2 and 3 AC components of the pressure signal fluctuate along the entire body, being strengthened in some locations, and weakened in others. In terms of the phase, a shift is observed in the *k* = 1 AC components in pressure- and shear-signals: the *k* = 1 AC pressure-signal of the posterior part generally shifts to a delayed phase, while the *k* = 1 AC shear-signal of the posterior part shows a fluctuation of phase modification. These signal changes concentrate on the neighbor-ward side of the body. On the free-side, the signal modification is insignificant.

On the contrary, when the protagonist fish is located in the front diagonal of the neighbor fish (see Figure 6, RHS), the protagonist fish senses very minor changes in both pressure- and shear-components, The slight change concentrates in the posterior body, still suggesting a possibility to sense a fish behind. The phase of *k* = 1 AC pressure- and shear-signals hardly have any notable change.

### Right Behind a Frontal Neighbor

**FIGURE 7:**
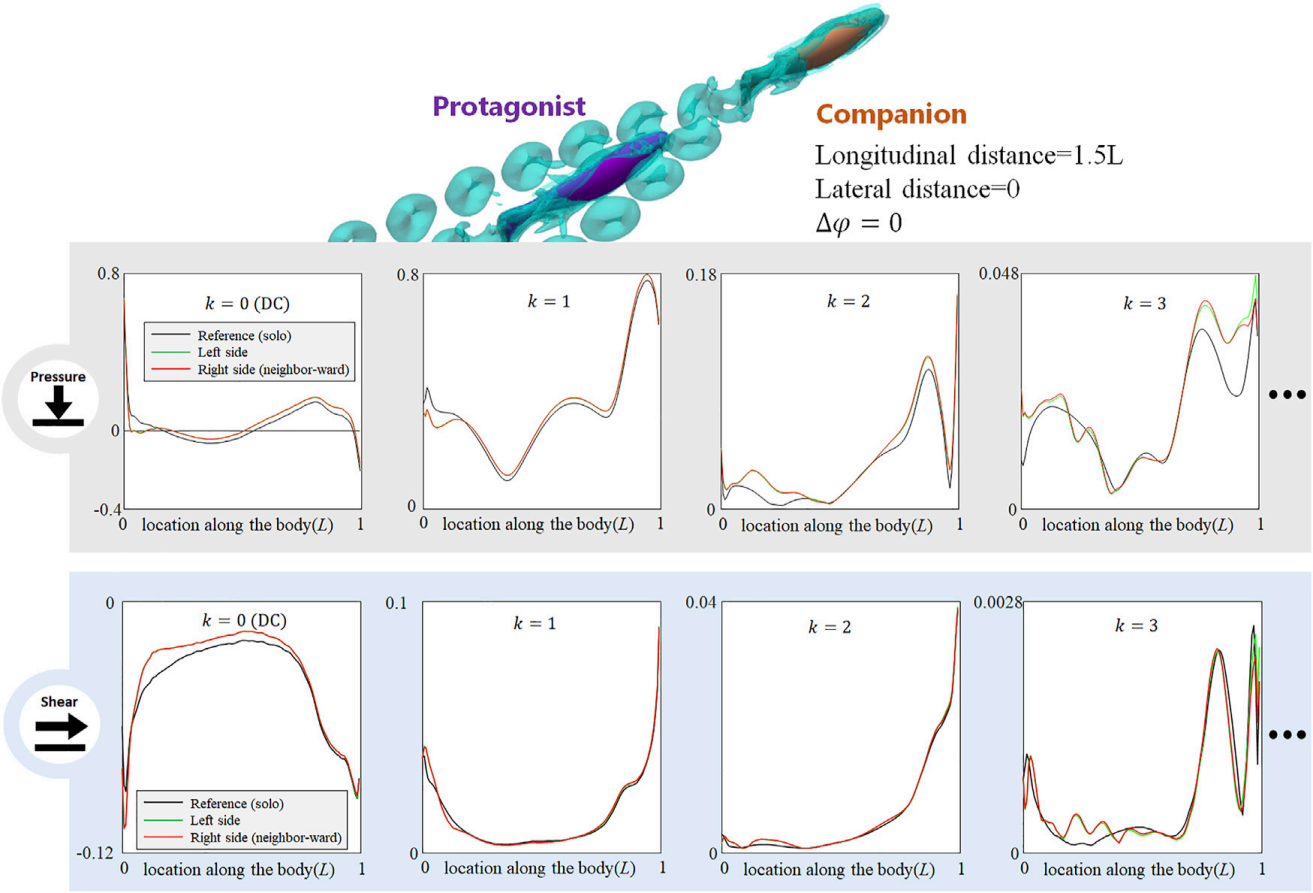
Signal features sensed by the protagonist fish when an in-phase neighbor is in front. Full results are presented in Supplementary Figure S10, ESM. One stress unit = 10 Pa.

When the neighbor fish is right in front of the protagonist fish, the DC pressure signal in the head zone of the protagonist fish decreases dramatically (e.g., DC pressure at around 0.05 L almost vanished), and the *k* = 1 AC component also shows moderate decrement in the head zone (see Figure 7, pressure). The higher order pressure signals, *k* = 2 and *k* = 3 AC, undergo irregular fluctuations. The shear stress on the entire body is reduced. Meanwhile, irregular extra fluctuations are observed in all AC shear signals (see Figure 7, shear). When the protagonist fish is right behind a neighbor fish, the stress modification on the left- and right-side body is generally same, which is regarded as a feature to identify such symmetric configurations.

Since the frontally swimming fish forms a rear zone with slower flow speed (see Figure 8B in Li et al. (2019a)), the protagonist fish in this zone may confront relatively slower oncoming flow, thus its frontal pressure and overall shear stress all attenuate, which reduces both form drag and friction. Meanwhile, the relatively slow oncoming flow will reduce the local slip ratio (the ratio of local flow speed to the fixed body-wave speed) at the posterior body, and the posterior body may generate stronger propulsion, expressed as the increment of the DC and *k* = 1 AC pressure-stress components at the posterior body.

**FIGURE 8:**
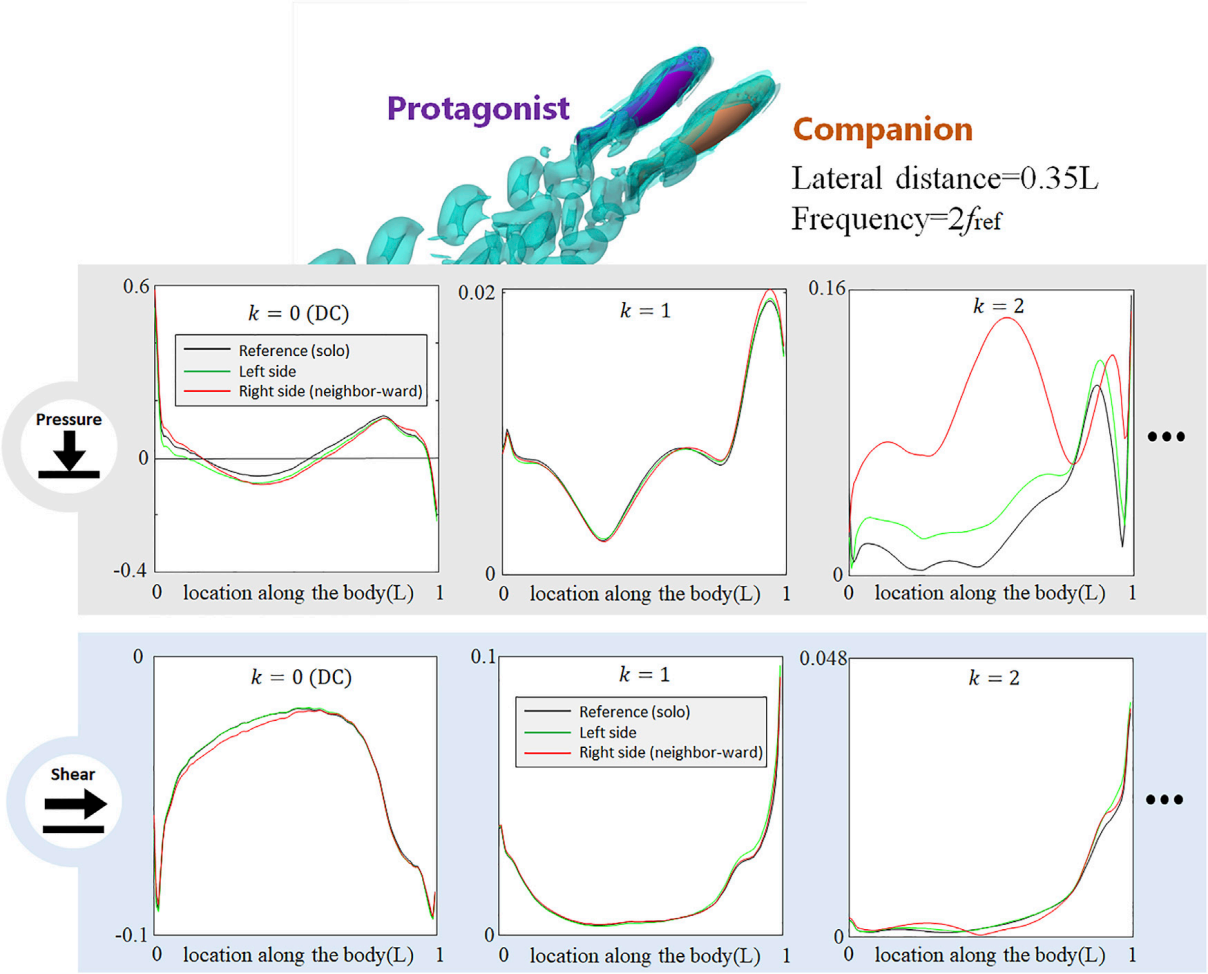
Signal features sensed by the protagonist fish when an alongside neighbor uses double frequency as the protagonist fish. Full results are presented in Supplementary Figure S11, ESM. One stress unit = 10 Pa.

### When the Neighbor Fish Uses a Different Tail-Beat Frequency

In all aforementioned simulations, both neighbor and protagonist fish use the same tail-beat frequency. In order to investigate if the changes in the tail-beat frequency of the neighbor fish can also be detected by the lateral-line stress signal, we doubled the tail-beat frequency of the neighbor fish with respect to that of the protagonist fish and simultaneously reduced the tail-beat amplitude to maintain a gross net-force balance. As shown in Figure 8, the doubled frequency used by the neighbor fish causes a dramatical change in the *k* = 2 AC pressure component, while the influences on *k* = 1 AC pressure component is much smaller than that observed in the case with the two fish using the same frequency (Figure 3). Interestingly, the DC pressure signal hardly reflects the change of the tail-beat frequency of the neighbor fish, retaining a similar pattern to that in Figure 3. As to the shear-stress signal, all components are insignificantly influenced. The shift of the AC pressure signal magnitude from *k* = 1 to *k* = 2 component suggests that the frequency of the neighbor fish can be sensed according to the frequency domain analysis. In contrast, DC signals of pressure and shear stress seem robust against a tail-beat frequency change of the neighbor fish.

## Discussion

This study provides a unique approach to understand the stress signals sensed by a fish lateral line. Based on Fourier Transform, the magnitude and phase in the frequency domain show characteristics that may be used to identify the relative position, phase and other status of a neighbor fish, even in the absence of a visual signal. It is worth to note that stress signals in the solo vs pair conditions to a large extent coincide with each other, and whether their difference can be distinguished by a fish or robot should still depend on a sufficiently accurate sensory system. Previous observations by Kanter and Coombs (Kanter and Coombs, 2003) suggest that the fish sensory system is rather accurate and robust to background flow where fish typically swim. They found that sculpin (*Cottus bairdi*) were able to detect relatively weak, prey-like signals in the presence of a strong ambient background flow, and further estimated that fish may sense flow changes several orders of magnitude below the background flow levels. The sensory system is also reported to concentrate at locations where changes in pressure are greatest during motion (Ristroph et al., 2015) to further strengthen sensing accuracy. In this study, the characteristic stress difference caused by a neighbour fish is usually one order of magnitude below the basal stress. Therefore, the sensitivity of a fish lateral line system, according to the literature, seems enough to detect the difference. Furthermore, since the water movements caused by a specific animal species usually show unique features (e.g., see Figure 1 in Mogdans (2019)), schooling fish could be instinctively sensitive to the characteristic signal formed by a neighbour fish of the same species, and less interfered by environmental noise.

Overall, the DC component of shear and pressure stresses can be used to judge the lateral distance of an alongside neighbor, while the magnitude and phase of the *k* = 1 AC pressure component can reflect the phase of an alongside neighboring fish. Similar to the pressure signal, the shear stress signal on the neighbor-ward side will change and indicates the lateral distance of the neighbor, but unlike the pressure signal, the shear signal is robust to the phase difference between the two fish (see Figure 5), and can provide a clear signal when the fish enters the wake of another fish (see Figures 6, 7). Since multiple vortices are generated during one tail-stroke, and further break down into small vortices, the wake flow causes lateral-line stress fluctuations with a frequency higher than the tail-beat frequency. Exposure to the wake of the neighbor cause changes in *k* ≥ 2 components, which may be sensed at the posterior part of the body or on the entire body, depending on the extent of the interaction between the protagonist fish with the wake of the neighbor fish. Through Fourier Transform, the tail-beat frequency of the neighbor fish can also be unveiled in the frequency domain—signal component corresponding to the neighbor fish tail-beat frequency will change sharply. The asymmetry between left- and right-side lateral-line signals can easily reflect on which side the neighbor is located. Our study only includes several basic cases in the vast state space of a two-fish system, and the characteristic signal modifications in more complex configurations remain to be examined by further investigation. Nevertheless, the present results give a useful quantitative assessment of the kind of signals that need to be processed by an effective lateral-line sensing system.

Regarding the influence caused by the phase difference in a fish pair, we only discussed one specific relative position ( ΔX=0.35L, ΔY=0L ). In this specific case, the pressure and phase signals at various Δφ show a mild change in magnitude, while their profiles are basically stable (see Figure 5). One should note that the change is moderate in this case because the longitudinal distance is zero, and the fish do not interact directly with each other’s wake structure. Previous experimental observation by Zheng et al. (2019) shows that when both phase-difference and longitudinal distance are present, the artificial lateral line can sense changes in the pressure signal, which suggests the phase difference can cause more significant stress change via wake vortex interaction.

There are limitations due to the numerical approach used in this study. Firstly, we simulated laminar flow, thus turbulence formed on the body surface and during the breakdown of the wake are beyond our simulation. Turbulence may disturb the lateral-line signal (Coombs et al., 1999), especially the higher terms in our analysis (*k* ≥ 2). Also, as the hydrodynamic scale increases, the boundary layer over the fish may become thinner, causing quantitative or even qualitative change in the surface stress. Secondly, the center of mass (CoM) in this research is fixed, therefore the perturbation effect on the CoM caused by the neighbor fish is neglected. Unlocking the CoM will produce a perturbation that may cause extra surface stress change. In addition, some modeling errors are associated with our numerical approach, such as 1) at the snout (about 0–0.05 L) and tail-tip (about 0.95∼1 L), there was some strong numerical fluctuation resulting from sharp corners. Similar numerical issues are also mentioned by Verma et al. (2019); 2) in the multi-block mesh system, the inter-block communication was realized by numerical interpolation, which might cause slight smoothing effect at the block interface.

It remains to be rigorously confirmed whether the sensing and nervous system of fish can process stress signals effectively filtering frequencies in a similar fashion to the Fourier Transform used in our study. The experimental-numerical study by Van Trump and McHenry, (2008) reports a 30-fold range in the amplitude of sensitivity and more than a 200-fold range of variation in cut-off frequency in lateral line sensing in zebrafish larvae (*Danio rerio*), which suggests that natural variation in cupular height within a species is capable of generating large differences in their mechanical filtering and dynamic range. On the other hand, even if a biological sensory system does not really perform FFT decomposition, we consider that this approach is especially suitable to engineering systems such as swimming robotics. Our study reminds that the stress signal sensed by an artificial lateral line should not be simply processed by averaging, and that the signal oscillating components carry useful information and should not be discarded. FFT is suitable for analyzing unsteadiness: high-frequency turbulence, low frequency ambient currents and self-induced flow can all be processed using FFT, leaving important DC and low-order AC components to indicate the state of a neighbor fish. Also, FFT can be implemented in artificial electronical systems with high efficiency. The stress signal regulation detected by FFT in this study must be applied flexibly in robotic applications, since robotic swimmers with variable morphological and kinematical properties may not possess universal signal features. We recommend researchers to implement preliminary tests to construct a database to connect the FFT stress-signal features with group status for their own robot swimmers, so that by comparing to the database, the regulation detected by FFT processing can be used to judge the schooling status in robot fish and assist to maintain group formation.

## Data Availability

The original contributions presented in the study are included in the article/Supplementary Material, further inquiries can be directed to the corresponding author.
