# Supplementary material for: Hydrodynamical Fingerprint of a Neighbour in a Fish Lateral Line
Source: Front Robot AI. 2022 Feb 11;9:825889. doi: 10.3389/frobt.2022.825889 (PMC8878980; doi:10.3389/frobt.2022.825889)
Supplement: Supplementary file 1 [file DataSheet1.PDF]

Electronic Supplementary Materials

Tittle: Hydrodynamical Fingerprint of a Neighbour in a Fish Lateral Line
Original research articles for Front. Robot. AI
Under Research Topic: Robotics to Understand Animal Behaviour

Gen Li [*1], Dmitry Kolomenskiy[2], Hao Liu[3], Benjamin Thiria[4], Ramiro Godoy-Diana[4]

1 Japan Agency for Marine-Earth Science and Technology (JAMSTEC), Japan
2 Skolkovo Institute of Science and Technology, Russia
3 Chiba University, Japan
4 Laboratoire de Physique et Mécanique des Milieux Hétérogènes (PMMH), CNRS UMR 7636, ESPCI Paris—PSL University, Sorbonne Université, Université de Paris, France

**Correspondence: G.L. (email: ligen@jamstec.go.jp)*

Part A Definitions of Symbols, Abbreviations and Parameters 2
Part B Additional Results 4
Part C Computational Model Information 15
C-1 Computational grids 15
C-2 Body length correction algorithm 16
C-3 Fluid solution 17
C-4 Boundary conditions and inter-block communications 19
C-5 Fast Fourier Transform (FFT) 20
Reference 21

Part A

Definitions of Symbols, Abbreviations and Parameters

Symbols and abbreviations	Physical meaning
A	tail-beat amplitude
AC	alternative current
CFD	computational fluid dynamics
CoM	centre of mass
$\Delta\varphi$	phase difference between two fish
ΔX	lateral distance between two fish
ΔY	longitudinal distance between two fish
DC	direct current of signal
f	frequency
f_{ref}	reference frequency
FT	Fourier transform
FFT	fast Fourier transform
k	dimensionless frequency, k=f/f_{ref}. k=1 component corresponds to tail-beat frequency, and k=0 corresponds to direct-current (DC) component.
L	length of the fish model, used as reference length
l	dimensionless distance from the snout along the longitudinal axis, $l \in [0,1]$
m	mass of fish model
Re	Reynolds number based on body length and swimming speed
t	time
t_P	period
U	cycle-averaged swimming speed
λ	length of the body wave
μ	viscosity of water
ρ	density of water

Table S2. Reference values.

**Parameter**	**Value**
density of water (ρ_{w})	1.00×10^{3} kg•m^{-3}
viscosity of water (μ_{w})	8.71×10^{-4} kg•m^{-1}•s^{-1}
length of fish (L)	4.0×10^{-2} m
reference frequency (f_{ref})	8 Hz
reference speed (U_{ref})	0.095 m•s^{-1}

Part B Additional Results

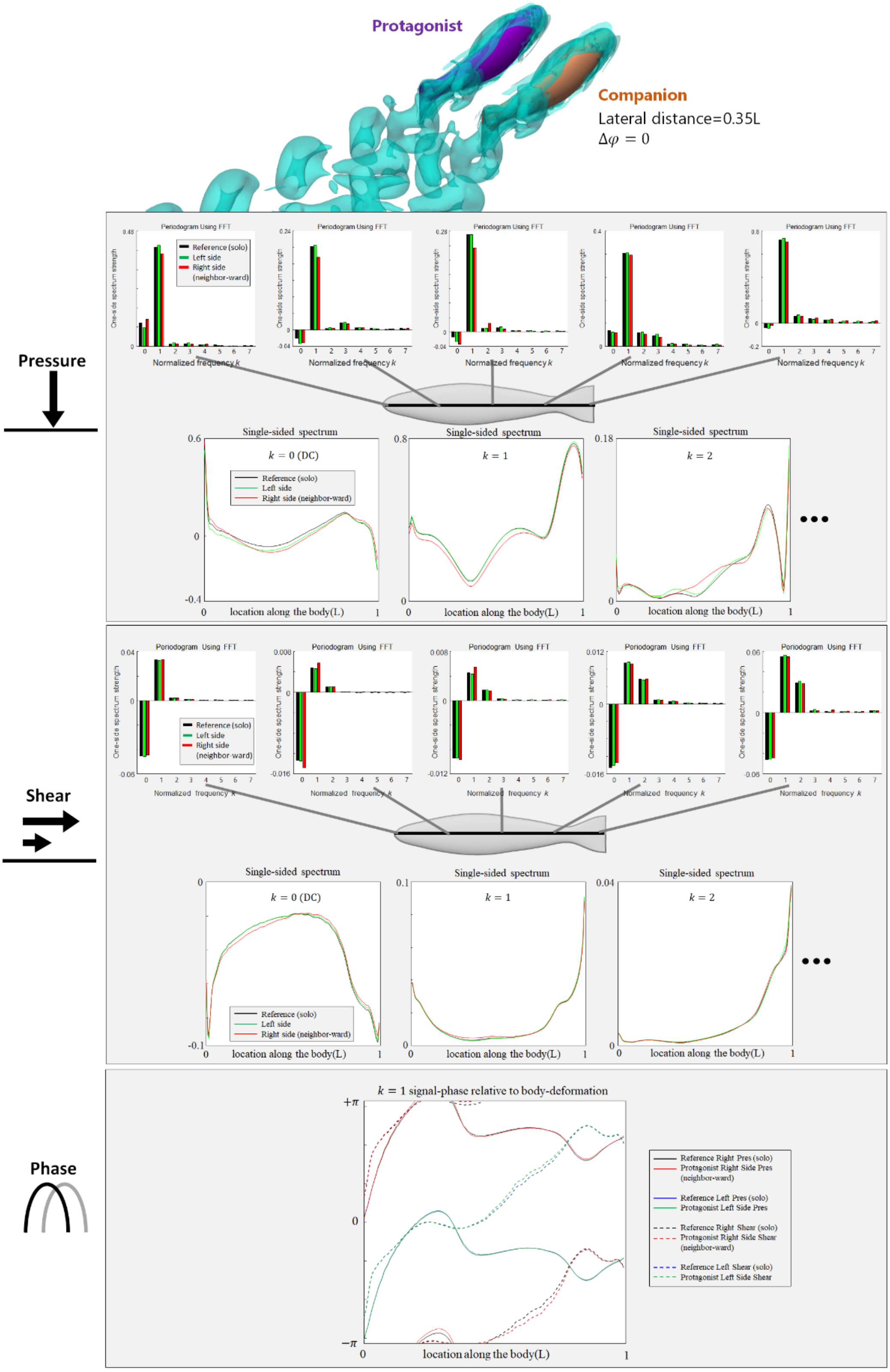

Fig. S1 Results of frequency domain analysis for lateral line sensing when a neighbor fish swim at lateral distance 0.35L and $\Delta\varphi = 0$ (in-phase). One stress unit=10 Pa.

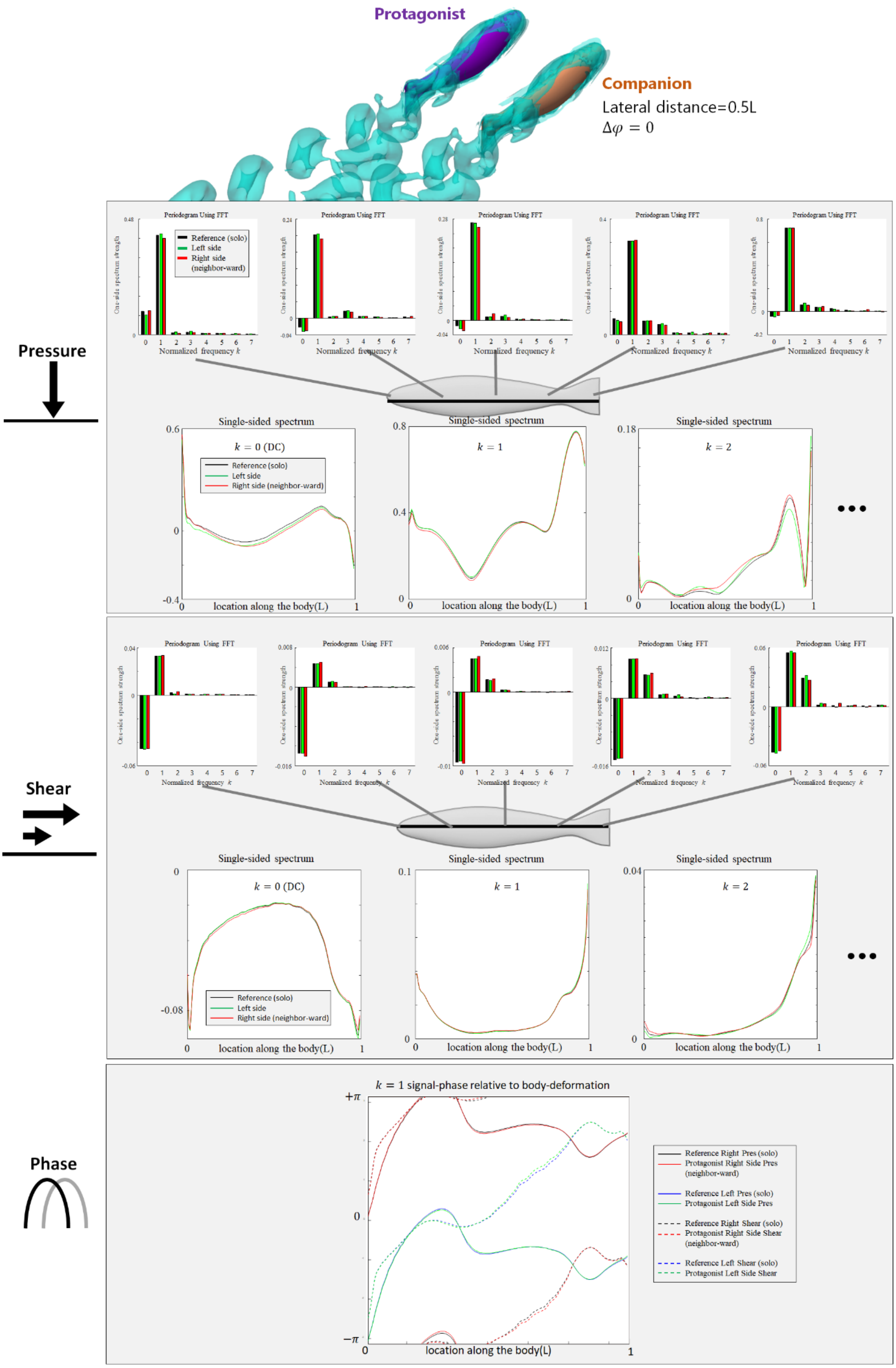

Fig. S2 Results of frequency domain analysis for lateral line sensing when a neighbor fish swim at lateral distance 0.5L and $\Delta\varphi = 0$ (in-phase). One stress unit=10 Pa.

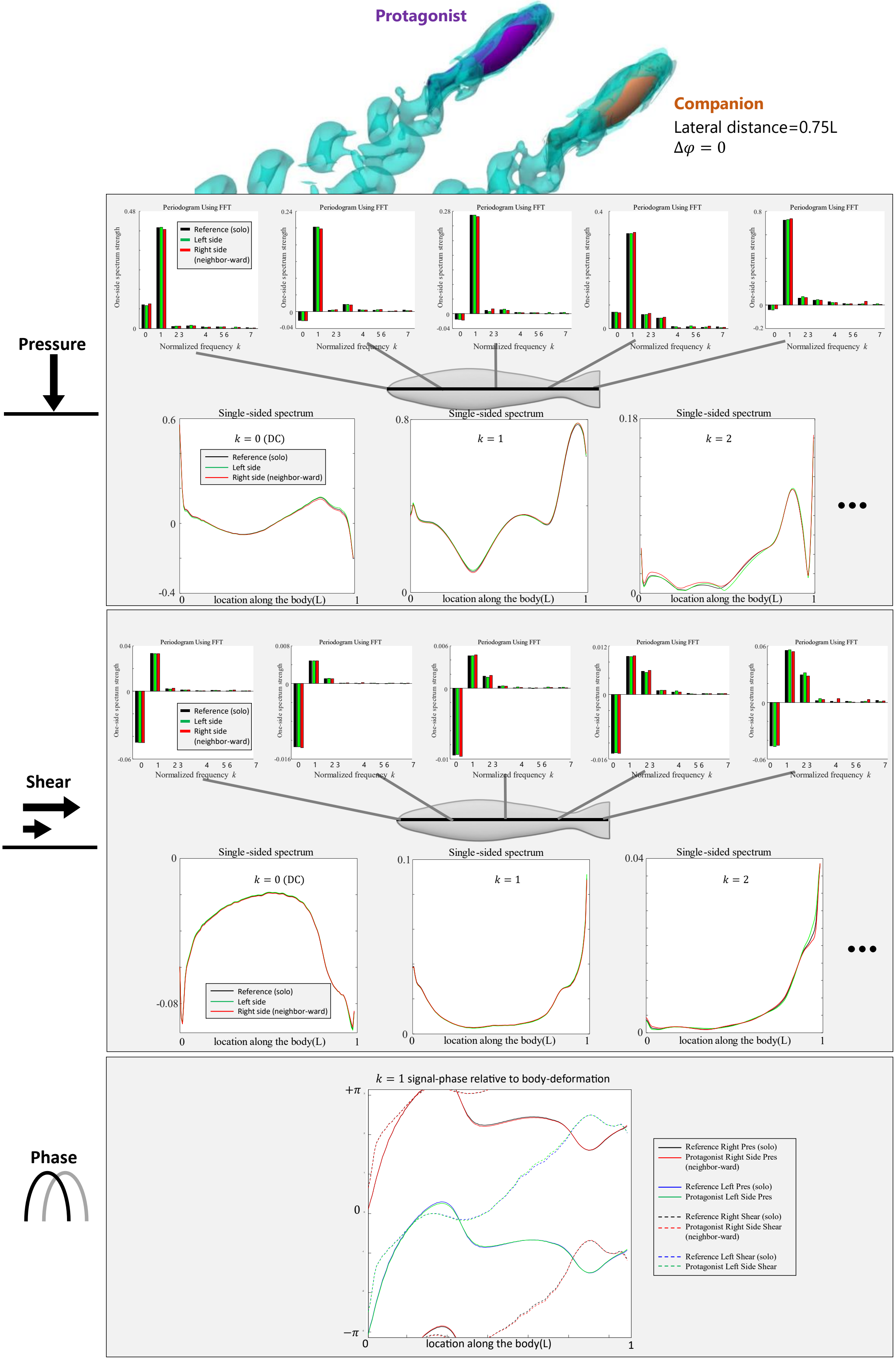

Fig. S3 Results of frequency domain analysis for lateral line sensing when a neighbor fish swim at lateral distance 0.75L and $\Delta\varphi = 0$ (in-phase). One stress unit=10 Pa.

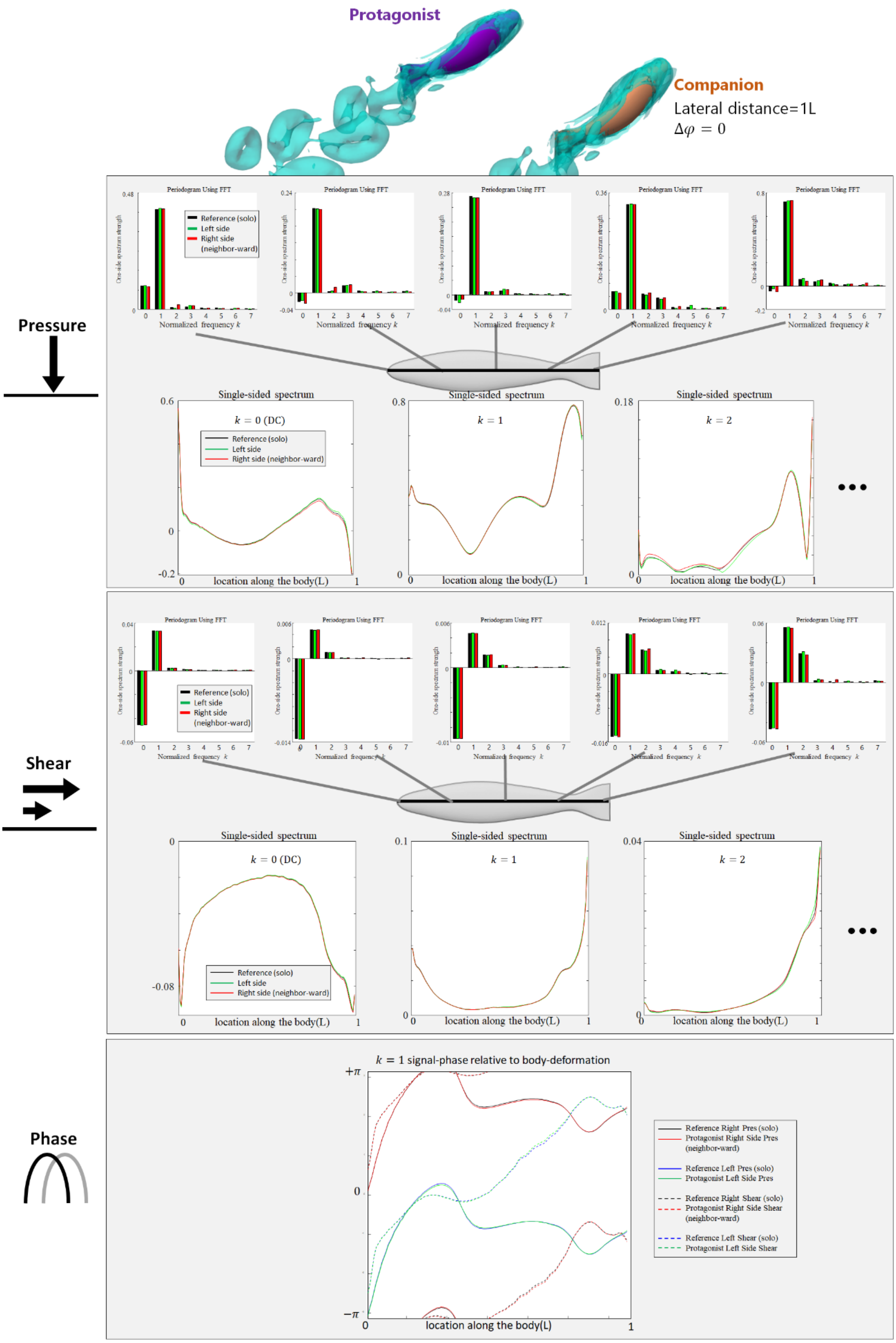

Fig. S4 Results of frequency domain analysis for lateral line sensing when a neighbor fish swim at lateral distance 1L and $\Delta\varphi = 0$ (in-phase). One stress unit=10 Pa.

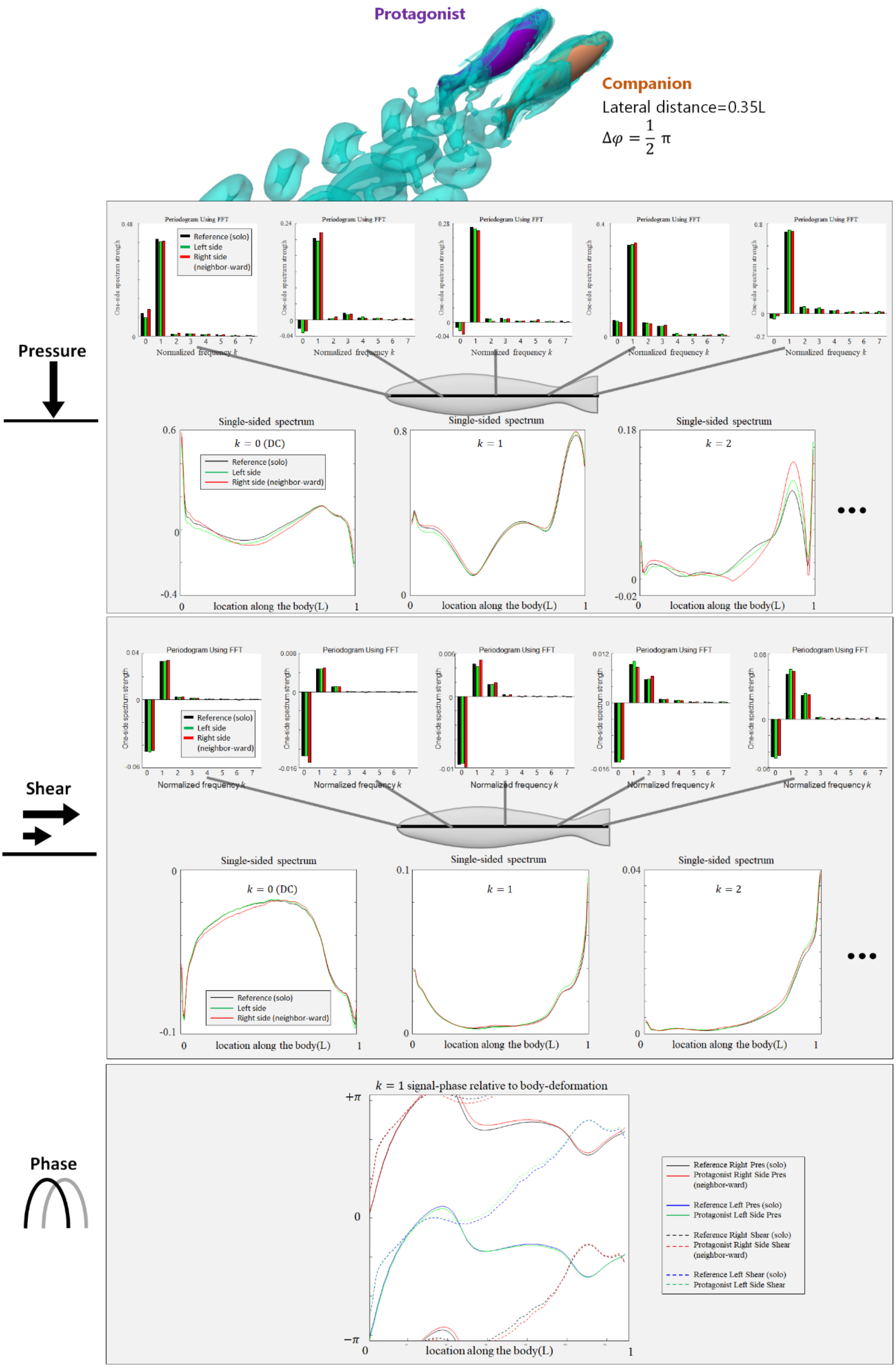

Fig. S5 Results of frequency domain analysis for lateral line sensing when a neighbor fish swim at lateral distance 0.35L and $\Delta\varphi = \frac{1}{2}\pi$. One stress unit=10 Pa.

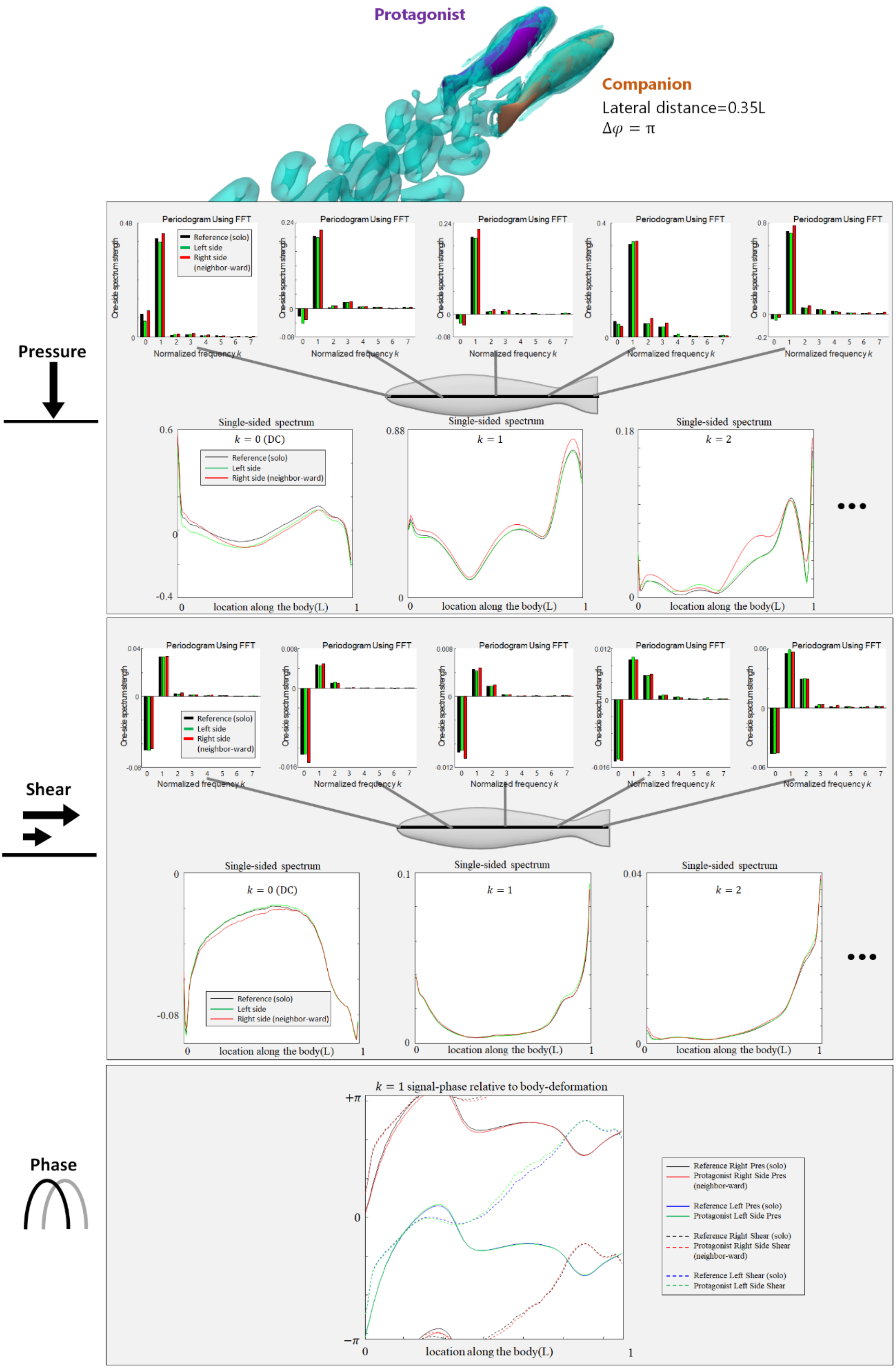

Fig. S6 Results of frequency domain analysis for lateral line sensing when a neighbor fish swim at lateral distance 0.35L and $\Delta\varphi = \pi$. One stress unit=10 Pa.

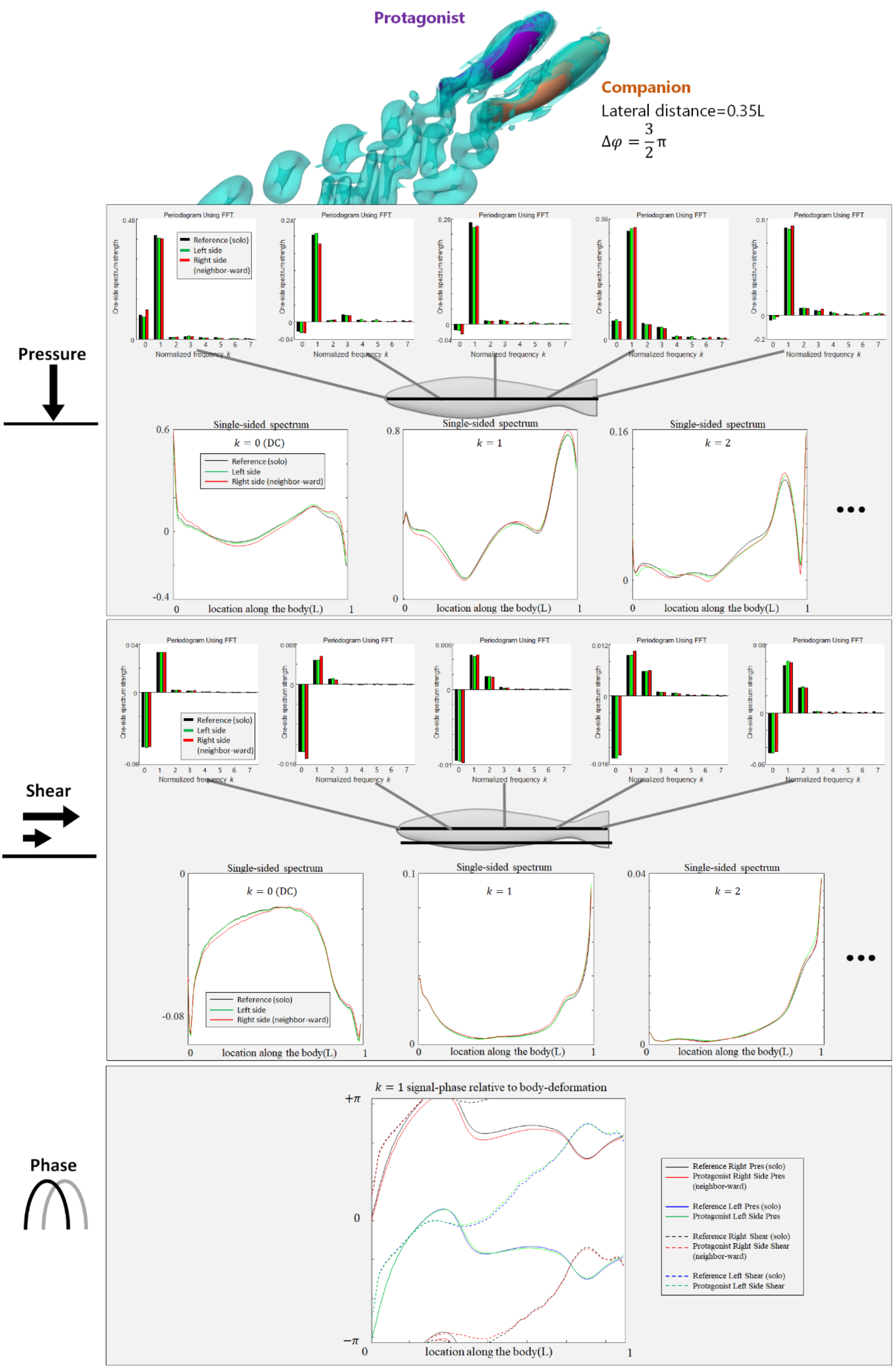

Fig. S7 Results of frequency domain analysis for lateral line sensing when a neighbor fish swim at lateral distance 0.35L and $\Delta\varphi = \frac{3}{2}\pi$. One stress unit=10 Pa.

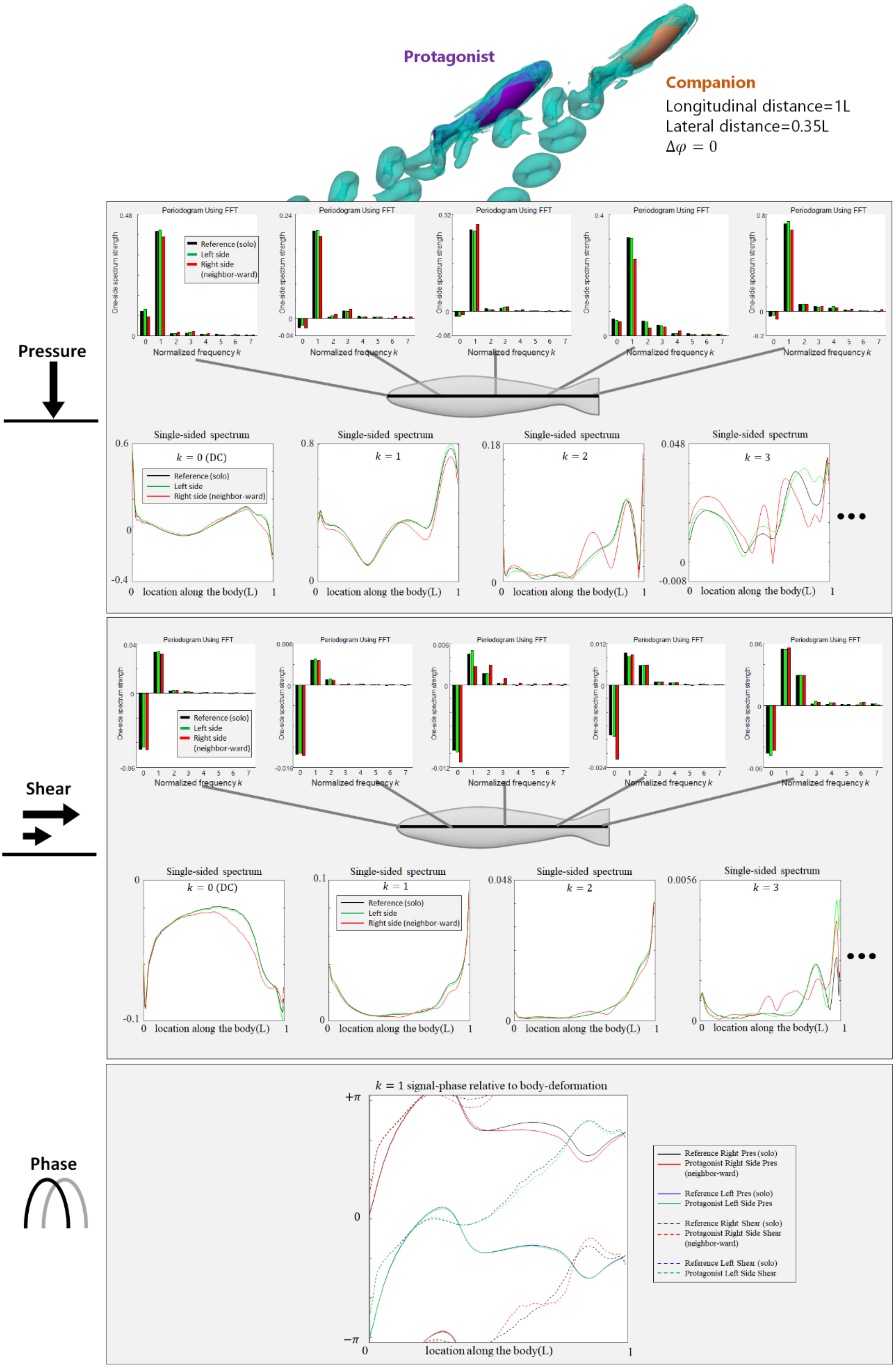

Fig. S8 Results of frequency domain analysis for lateral line sensing when a neighbor fish swim in diagonal front. One stress unit=10 Pa.

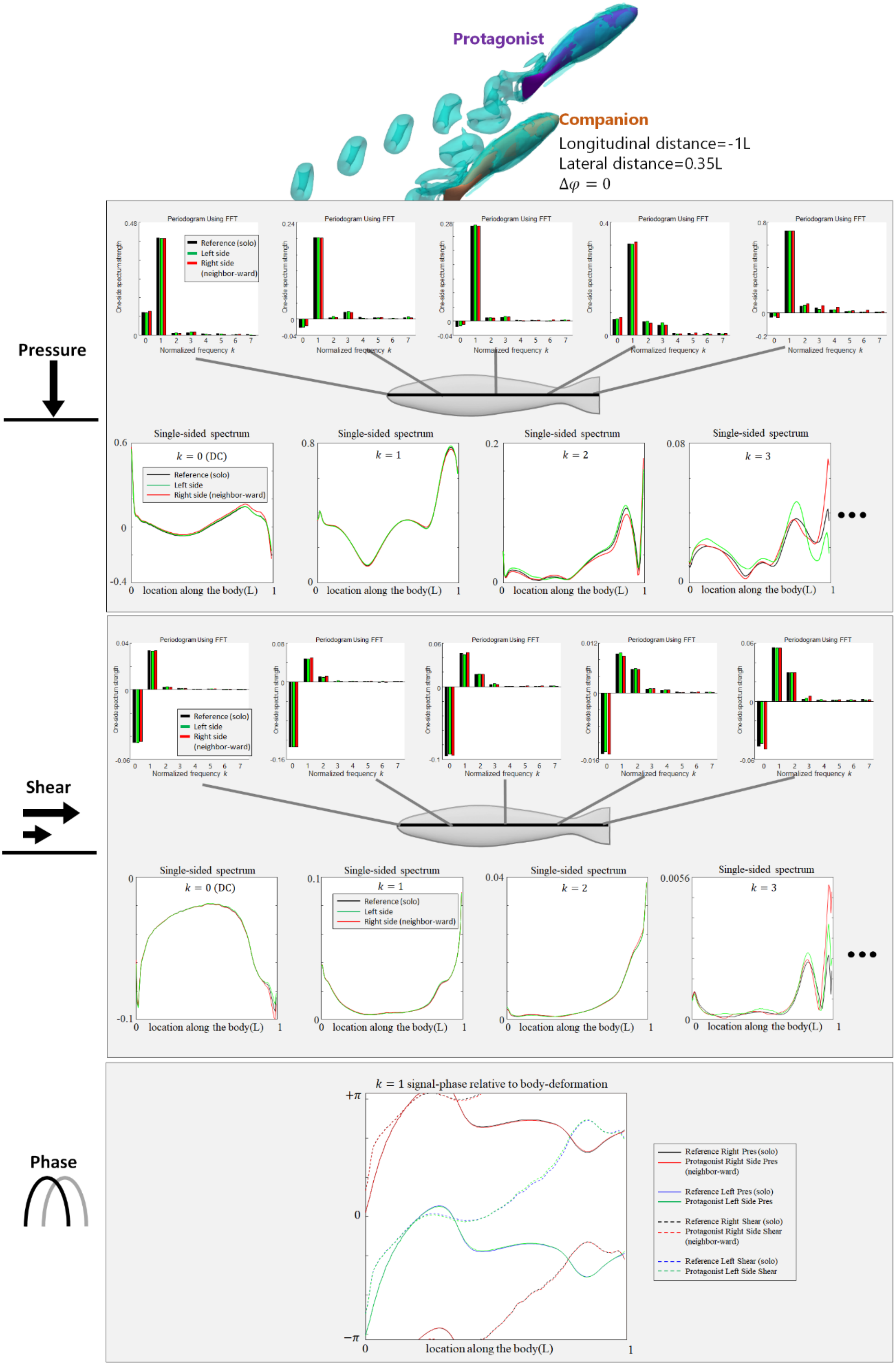

Fig. S9 Results of frequency domain analysis for lateral line sensing when a neighbor fish swim in diagonal rear. One stress unit=10 Pa.

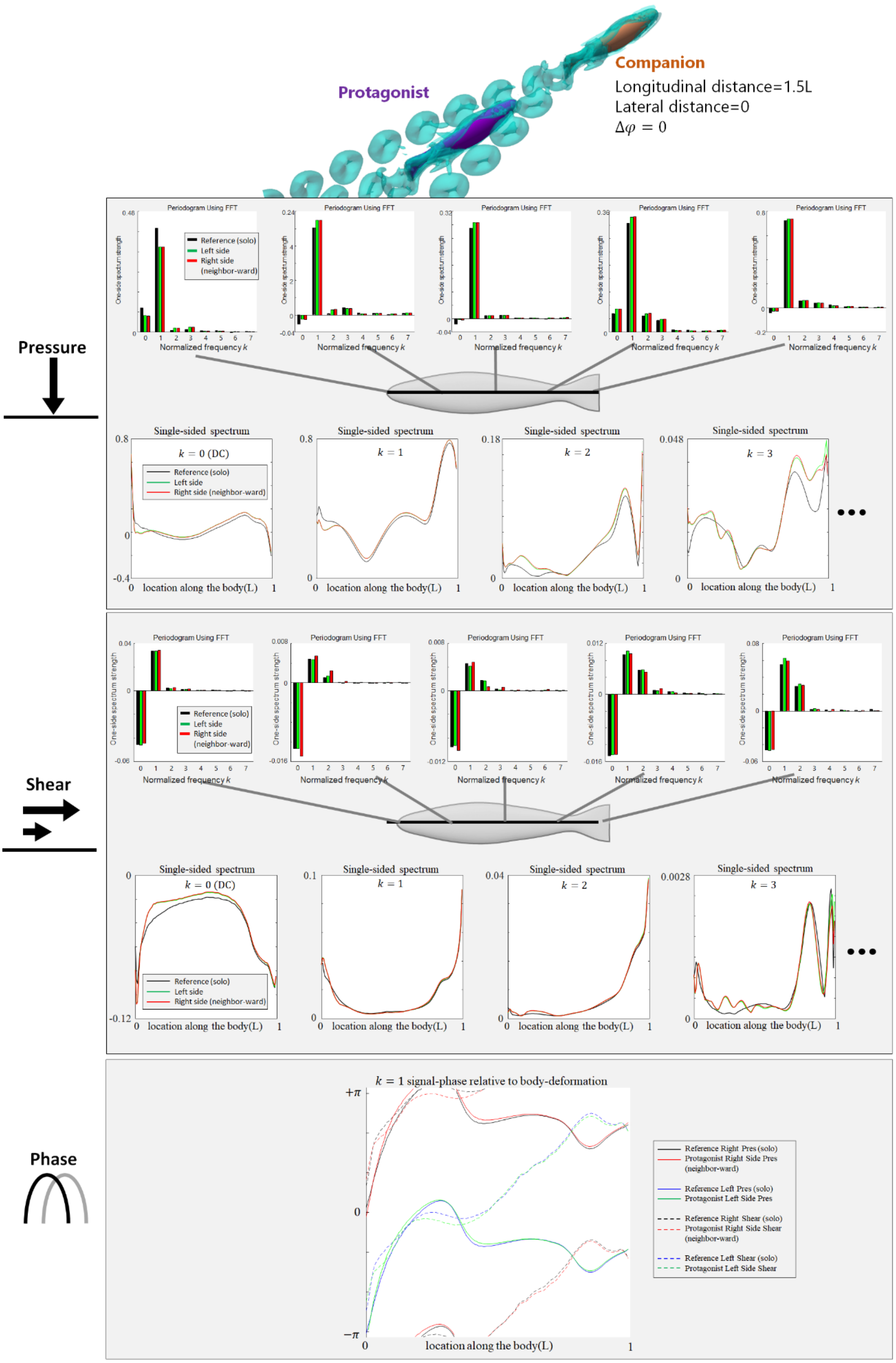

Fig. S10 Results of frequency domain analysis for lateral line sensing when a neighbor fish swim in front. One stress unit=10 Pa.

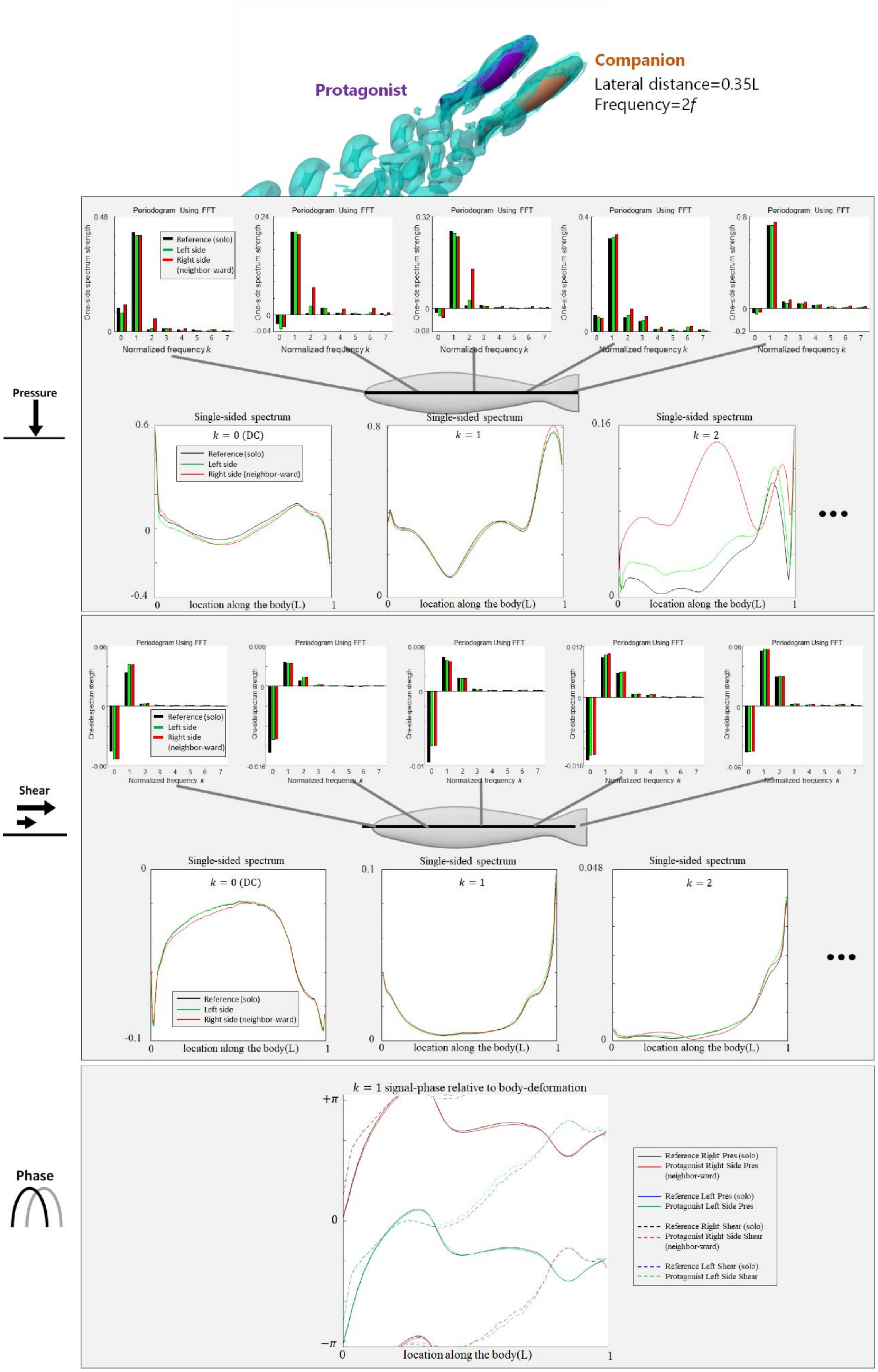

Fig. S11 Results of frequency domain analysis for lateral line sensing when an alongside neighbor fish swim with doubled frequency. One stress unit=10 Pa.

Part C Computational Model Information

C-1 Computational grids

As shown by Fig.S12, the computational model is a multi-blocked, overset-grid system comprising a body-fitted grid and a global grid to model the flow patterns generated by the fish with sufficient resolution both in the near and the far field. The innermost surface of the body-fitted grid represents the surface model of fish. The radial width of body-fitted grid was defined to be less or equal to one third of the body length of the fish model. The body grid had 20 radial layers, with the radial width of the innermost layer adjacent to the body surface defined to be $\leq 0.1L/\sqrt{\mathrm{Re}}$, an empirical formula ensuring that the grid resolution near the fish body is suitably accurate for the flow condition, where L is the body length of the fish and Re is the Reynolds number. Furthermore, the radial width of the body-fitted grid at each time step was co-determined by body curvature to accommodate the strong body deformations—radial width varied in order to avoid overlap between nearby grid cells. To simulate the flow around the fish, the body-fitted grid was updated at each time step. In previous studies (Li et al., 2012, 2014, 2016), we verified the necessary density (the number of cells per unit space) of the body-fitted grid. The global grid possesses a similar density as the outer layer of the body-fitted grid where the two grids overlap. To avoid artefacts at the external boundaries of the global grid on the solution around the fish, we performed a sensitivity analysis in our previous study (Li et al., 2016) to ensure that we used a sufficiently large computational domain.

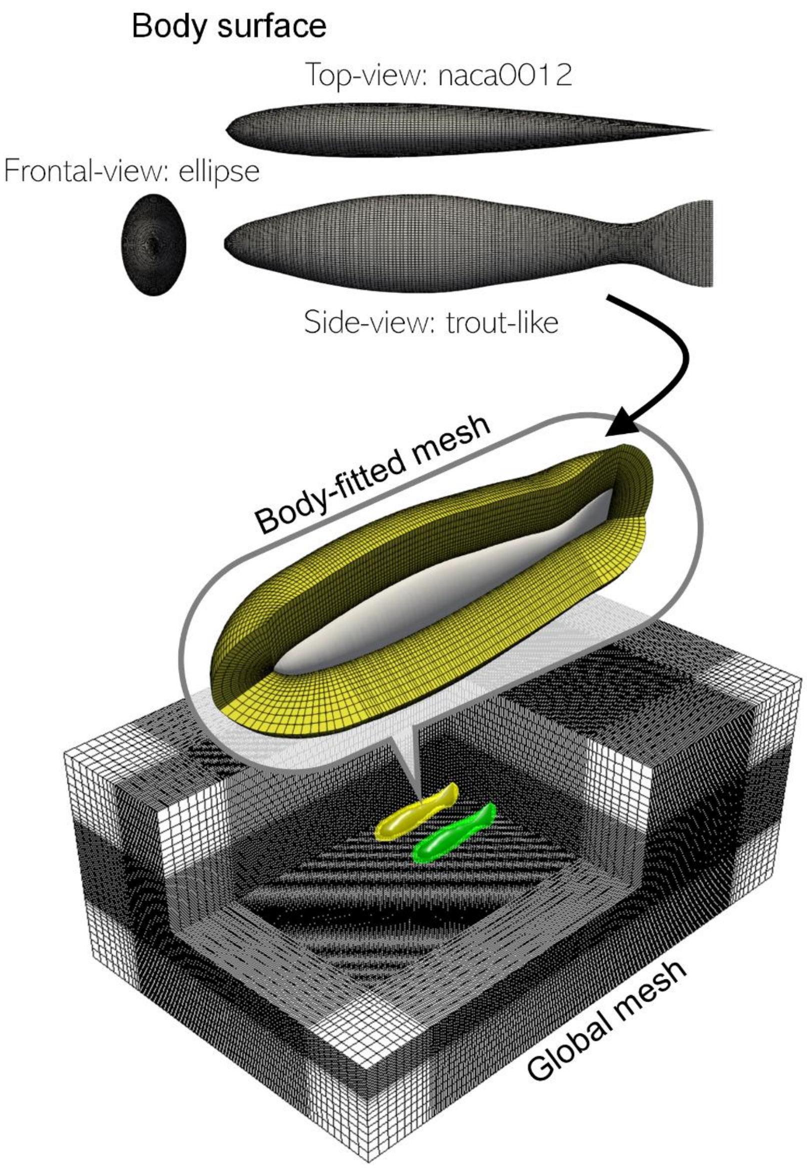

Figure S12. Computational grid system. A multi-blocked, overset-grid system comprises a body-fitted mesh (yellow) generated from surface model (97×133), dimension for simulations: 97×133×20, and a global mesh (white), which is taken sufficiently large to allow accurate simulations of the fish in terms of wake topology.

C-2 Body length correction algorithm

The sinusoidal functions driving the deformation fish causes the total body length along the midline to vary during the tail beat. This variation is corrected at every time step by a procedure that preserves the lateral excursion while ensuring that the body length remains constant. figure S13 explains the procedure of this correction:

An axis (green axis in figure S13) is first generated according to the sinusoidal function. The length of this axis exceeds the actual body length. Then, in the longitudinal direction the green axis is linearly contracted until it reaches the actual length, while the lateral excursion is preserved. The corrected axis is used as the body shape of the fish in the simulation at this time step.

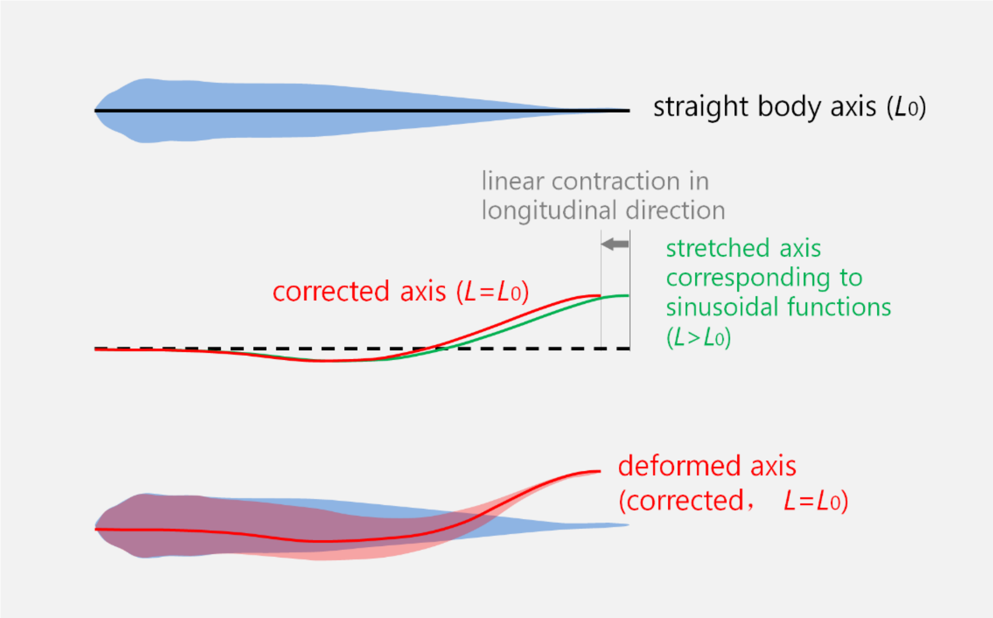

Figure S13. Body length variation caused by deformation is corrected by a procedure that preserves the lateral excursion while ensuring a constant body length.

C-3 Fluid solution

The fluid module provides fortified solutions to the three-dimensional Navier-Stokes (NS) equations in an inertial frame of reference. The solving process is implemented using the finite volume method (FVM), based on a multi-block, overset mesh system and inter-block communication algorithm.

The governing equations for the fluid solution are the three-dimensional, incompressible and unsteady NS equations written in strongly conservative form for mass and momentum (Liu, 2009). To accelerate the computation and improve the robustness during iteration, the artificial compressibility method is adopted by adding a pseudo time derivative of pressure to the continuity equation (Liu, 2009). The governing equations for an arbitrary deformable control volume V are

$$\int_V \left(\frac{\partial \mathbf{Q}}{\partial t} + \frac{\partial \mathbf{q}}{\partial \tau}\right) dV + \int_V \left(\frac{\partial \mathbf{F}}{\partial x} + \frac{\partial \mathbf{G}}{\partial y} + \frac{\partial \mathbf{H}}{\partial z} + \frac{\partial \mathbf{F_v}}{\partial x} + \frac{\partial \mathbf{G_v}}{\partial y} + \frac{\partial \mathbf{H}}{\partial z}\right) dV = 0$$

where

$$\mathbf{Q}=\begin{bmatrix}u\\v\\w\\0\end{bmatrix},\ \mathbf{q}=\begin{bmatrix}u\\v\\w\\p\end{bmatrix},\ \mathbf{F}=\begin{bmatrix}u^2+p\\uv\\uw\\\lambda u\end{bmatrix},\ \mathbf{G}=\begin{bmatrix}vu\\v^2+p\\vw\\\lambda v\end{bmatrix},\ \mathbf{H}=\begin{bmatrix}wu\\wv\\w^2+p\\\lambda w\end{bmatrix},$$

$$\mathbf{F_v}=-\frac{1}{Re}\begin{bmatrix}2u_x\\u_y+v_x\\u_z+w_x\\0\end{bmatrix},\ \mathbf{G}_v=-\frac{1}{Re}\begin{bmatrix}v_x+u_y\\2v_y\\v_z+w_y\\0\end{bmatrix},\ \mathbf{H}_v=-\frac{1}{Re}\begin{bmatrix}w_x+u_z\\w_y+v_z\\2w_z\\0\end{bmatrix},$$

and u, v, and w are velocity components respectively in X-, Y- and Z-directions of the Cartesian coordinate system; velocity components with subscripts x, y, and z are velocity gradients respectively in X-, Y-, and Z-directions of the Cartesian coordinate system; p is the pressure; λ is the pseudo-compressibility coefficient; the set of equations modified from the incompressible Navier-Stokes equations can be solved implicitly by marching in pseudo time: t denotes the physical time, τ denotes the pseudo time, and the term $\mathbf{q}$ associated with the pseudo time is designed for an inner-iteration inside each physical time step, and will vanish when the divergence of velocity is driven to zero so as to satisfy the equation of continuity. By introducing the Reynolds transport theorem and employing the Gauss integration theorem, an integrated form of the Navier-Stokes equations in general curvilinear coordinate system corresponding to the FVM structural mesh is gained as

$$\frac{\partial}{\partial t}\int_V \mathbf{Q}\,dV+\int_V\frac{\partial \mathbf{q}}{\partial \tau}dV+\oint_S(\mathbf{f}-\mathbf{QU}_V)\cdot\mathbf{n}\,dS=0\,,$$

Where $\mathbf{f}=(\mathbf{F}+\mathbf{F}_v,\mathbf{G}+\mathbf{G}_v,\mathbf{H}+\mathbf{H}_v,)$; the control volume V is a hexahedral cell, while S denotes the surfaces of the hexahedral cell; $\mathrm{n}=\left(n_x,n_y,n_z\right)$ is the unit outward normal vector corresponding to all the surfaces of the hexahedral cell; $\mathbf{U}_V$ is the local velocity of the moving cell surface caused by displacement and deformation of the cell. For a structured, three-dimensional mesh system (ξ-, η-, and ζ-dimensions respectively represent each of the three dimensions of mesh) and cell-centered storage architecture, A semi-discreate form can be further derived:

$$\frac{\partial}{\partial t}(\mathbf{Q}V)_{ijk}+\left(\frac{\partial \mathbf{q}}{\partial \tau}\right)_{ijk}V_{ijk}+\mathbf{R}_{ijk}=0\,,$$

where

$$\mathbf{R}_{ijk}=\left(\hat{\mathbf{F}}+\hat{\mathbf{F}}_v\right)_{i+\frac{1}{2},j,k}-\left(\hat{\mathbf{F}}+\hat{\mathbf{F}}_v\right)_{i-\frac{1}{2},j,k}+\left(\hat{\mathbf{G}}+\hat{\mathbf{G}}_v\right)_{i,j+\frac{1}{2},k}-\left(\hat{\mathbf{G}}+\hat{\mathbf{G}}_v\right)_{i,j-\frac{1}{2},k}$$
$$+\left(\hat{\mathbf{H}}+\hat{\mathbf{H}}_v\right)_{i,j,k+\frac{1}{2}}-\left(\boldsymbol{H}+\hat{\mathbf{H}}_v\right)_{i,j,k-\frac{1}{2}}$$

$$\hat{\mathbf{F}}+\hat{\mathbf{F}}_v=(\mathbf{f}-\mathbf{QU}_V)\cdot\mathbf{S_n},\ \hat{\mathbf{G}}+\hat{\mathbf{G}}_v=(\mathbf{f}-\mathbf{QU}_V)\cdot\mathbf{S_n},\ \hat{\mathbf{H}}+\hat{\mathbf{H}}_v=(\mathbf{f}-\mathbf{QU}_V)\cdot\mathbf{S_n},$$
$$\mathbf{S_n}=\left[S_{\mathrm{nx}},S_{\mathrm{ny}},S_{\mathrm{nz}}\right]=\mathbf{n}\cdot S$$

i, j, and k are cell indexes respectively in ξ-, η-, and ζ-dimensions in the boundary-fitted curvilinear coordinate system, while "$+\frac{1}{2}$" and "$-\frac{1}{2}$" denote the surface location (e.g. $i+\frac{1}{2}$ denotes a surface locates on the positive ξ-direction of the hexahedral cell V); $\hat{\mathbf{F}}$, $\hat{\mathbf{G}}$ and $\hat{\mathbf{H}}$ denote inviscid flux in ξ-, η-, and ζ-directions, respectively; $\hat{\mathbf{F}}_v$, $\hat{\mathbf{G}}_v$ and $\hat{\mathbf{H}}_v$ denote viscous flux in ξ-, η-, and ζ-directions, respectively; For each surface of the hexahedral cell, $\mathbf{S_n}$ denotes a vector consisting of projected areas of present surface in X-, Y-, and Z-directions, respectively; $\mathbf{n}$ is the unit outward normal vector of the present surface in the Cartesian coordinate system, and

S is the area of the present surface.

C-4 Boundary conditions and inter-block communications

The computational mesh consists of two blocks as shown in figure S12: one is surrounding the fish body, deforming and moving with the fish and constructed in a body-fitted curvilinear coordinate system (ξ-, η-, and ζ-dimensions are circumferential, axial and radial directions relative to the fish body, respectively); the other is a static background block, of which the ξ-, η-, and ζ-dimensions, respectively, overlap the X-, Y-, and Z-dimensions of the Cartesian coordinate system.

The NS equations are solved in each block. The boundary conditions for the NS equations are: 1) in the fish-body-fitted mesh, the non-slip condition is applied to the cells on the surface of the fish body; 2) in the global mesh, an incoming flow U is set for frontal surface, while zero-gradient condition is used for other surface; 3) at the interfaces between fish-body-fitted and global meshs, the two meshs provide boundary conditions to each other through interpolations.

Table S4. Locations of additional information regarding the computational approach.

specific information		relevant publication
methods	numerical solutions to Navier-Stokes equations	(Liu, 2009)
	multi-blocked, overset-grid system and inter-block interpolation	(Liu, 2009; Li et al., 2012)
	inter-body cell	(Li et al., 2016) (in supplementary materials)
validations	mesh density independence test	(Li et al., 2014) (in supplementary materials) Note: in this study the resolution based on grid density is higher than that in (Li et al., 2014)
	mesh size independence test	(Li et al., 2016) (in supplementary materials)
	validation on hydrodynamic solution on an oscillating cylinder, compared with experiment	(Li et al., 2014)(in supplementary materials)
	validation on flow field on swimming fish, compared with PIV	(Li et al., 2012, 2016)
	validation on motion solution on swimming fish, compared with experiment	(Li et al., 2012, 2014)

C-5 Fast Fourier Transform (FFT)

A frequency-domain representation includes a frequency-domain graph showing how much of the signal lies within each given frequency band over a range of frequencies, as well as the phase shift of each sinusoid, in order to be able to recombine the frequency components to recover the original time signal.

The lateral-line stress signal **X** during an arbitrary tail-beat cycle is sampled and processed by a fast Fourier transform (FFT) algorithm (function: *fft*, MATLAB R2020b, The Mathworks), which computes the discrete Fourier transform (DFT, (Frigo and Johnson 1998)) of signal vector **X**:

$$\mathbf{Y} = \mathrm{fft}(\mathbf{X})$$

where $\mathbf{Y} = (y_0, y_1, \ldots, y_{N-1})$. $y_0, y_1, \ldots, y_{N-1}$ are complex numbers that define the DFT formula

$$\mathrm{y}_k = \sum_{n=0}^{N-1} x_n e^{-i2\pi kn/N} \qquad k = 0,1,\ldots,N-1,$$

$e^{i2\pi/N}$ is a primitive Nth root of 1, N the signal length and k is the dimensionless frequency used in this study, computed by $k = f/f_{\mathrm{ref}}$, where the k=1 component corresponds to that in tail-beat frequency, and k=0 corresponds to the direct current component. We will use in the paper the direct current (DC), alternating current (AC) nomenclature.

The two-sided spectrum $\mathbf{P}_{\mathrm{two-side}}$ is computed as:

$$\mathbf{P}_{\mathrm{two-side}} = \left|\frac{\mathbf{Y}}{N}\right|$$

We show only the positive half of the frequency spectrum because the spectrum of the stress signal is symmetrical around the DC component ($f = 0$). The single-sided spectrum **P** is computed as:

$$P(k) = \mathrm{signum}(y_0) \cdot P_{\mathrm{two-side}}(k), \quad k = 0\ (DC)$$

$$P(k) = 2P_{\mathrm{two-side}}(k),\ \ k = 1 \text{ to} \frac{N}{2} - 1\ (AC)$$

Note that for the DC component, we preserve the sign of y_0 since the negativity of the DC component possesses a physical meaning (negative stress). The remainder of the two-sided spectrum $\mathbf{P}_{\mathrm{two-side}}$, $\frac{N}{2}$ to N, is discarded.

The phase of each component of **Y** is also computed (using the MATLAB function *angle*).

References

Frigo, M., and Johnson, S. G. (1998). FFTW: An adaptive software architecture for the FFT. *ICASSP, IEEE Int. Conf. Acoust. Speech Signal Process. - Proc.* 3, 1381–1384. doi:10.1109/ICASSP.1998.681704.

Li, G., Müller, U. K., Van Leeuwen, J. L., and Liu, H. (2012). Body dynamics and hydrodynamics of swimming fish larvae: A computational study. *J. Exp. Biol.* 215, 4015–4033. doi:10.1242/jeb.071837.

Li, G., Müller, U. K., Van Leeuwen, J. L., and Liu, H. (2014). Escape trajectories are deflected when fish larvae intercept their own C-start wake. *J. R. Soc. Interface* 11. doi:10.1098/rsif.2014.0848.

Li, G., Müller, U. K., Van Leeuwen, J. L., and Liu, H. (2016). Fish larvae exploit edge vortices along their dorsal and ventral fin folds to propel themselves. *J. R. Soc. Interface* 13. doi:10.1098/rsif.2016.0585.

Liu, H. (2009). Integrated modeling of insect flight: From morphology, kinematics to aerodynamics. *J. Comput. Phys.* 228, 439–459. doi:10.1016/j.jcp.2008.09.020.
